# The impact of urbanisation on social behaviour: a comprehensive review

**DOI:** 10.1002/brv.70113

**Published:** 2025-12-29

**Authors:** Avery L. Maune, Barbara A. Caspers, Isabel Damas‐Moreira

**Affiliations:** ^1^ Department of Behavioural Ecology Bielefeld University Konsequenz 45 Bielefeld 33615 Germany; ^2^ Joint Institute for Individualisation in Changing Environments University of Münster and Bielefeld University Bielefeld Germany

**Keywords:** city, social system, social organisation, social structure, mating system, care system, communication, interspecific interactions, anthropogenic change, urban stressor

## Abstract

Urbanisation is a key driver of global environmental change and presents animals with novel stressors and challenges. It can fundamentally influence social behaviour and has the potential to reshape within‐ and between‐species social interactions. Given the role of social behaviour in reproductive fitness and survival, understanding how social interactions change in response to urban conditions is crucial in addressing individual‐, population‐, and species‐level responses to urbanisation, as well as the consequential ecological impacts. Here, we conducted the first systematic review addressing the impact of urbanisation on social systems and interspecific interactions. We synthesise the outcomes of the 227 studies from our literature search, organised across three key topics: (*i*) effects of urban stressors on social behaviour (*N* = 170), (*ii*) social system responses to urban environments (*N* = 75), and (*iii*) the impact of urbanisation on interspecific interactions (*N* = 12). Our review revealed that urbanisation is having a substantial impact on multiple facets of social behaviour, with 92% of studies finding a significant impact. We also identified several biases and gaps in the current literature. For example, 62% of all studies were conducted on birds, and 85% of studies testing urban stressors focused on anthropogenic noise. Given the diversity of animal social systems, there is obvious variation in social responses to urban conditions. However, we offer predictions for how social systems might change as urban environments continue to expand rapidly and suggest guidelines for future research to enhance generalisations across taxa. Our review brings together multiple areas of research, provides timely insights and outlines a framework for a unified and proactive approach to addressing social responses to urbanisation. This represents an essential foundation for anticipating species' responses to urban expansion and guiding effective conservation efforts.

## I. INTRODUCTION

Urbanisation is rapidly expanding and is a well‐established global threat to wildlife and biodiversity (McKinney, 2006; Shochat *et al*., 2010). It creates fragmented habitats that are characterised by built‐up infrastructure, human activity, sensory and chemical pollutants, as well as changes in community composition relative to more natural habitats (Longcore & Rich, 2004; Grimm *et al*., 2008; Halfwerk & Slabbekoorn, 2015; Falchi *et al*., 2016; Basak *et al*., 2023; Carlon & Dominoni, 2024). Animals inhabiting urban areas face novel challenges and potentially experience environmental change at a more rapid timescale than in their previous evolutionary history (Sih, Ferrari & Harris, 2011). These so‐called ‘urban stressors’ can impose selection pressures that translate into behavioural modifications, such as changes in foraging, dispersal, habitat use, vigilance, and social behaviour, which allow animals to adjust quickly to changing environmental conditions (Tuomainen & Candolin, 2011; Lowry, Lill & Wong, 2013; Wong & Candolin, 2015). As urbanisation affects individuals and populations, and considering the impact anthropogenic change can have on social behaviour (Blumstein, Hayes & Pinter‐Wollman, 2023), it is only logical that cities can also deeply impact an animal's social system and influence the dynamics between species.

Social behaviour encompasses all interactions that occur between individuals (Blumstein *et al*., 2010) and is ubiquitous in the animal kingdom (Doody, Burghardt & Dinets, 2013). Social behaviour lies on different levels of complexity, influenced by a species' social system, which builds on four interrelated components: (*i*) social organisation, (*ii*) social structure, (*iii*) mating system, and (*iv*) care system (Kappeler, 2019). Local environmental conditions can influence social systems by altering the dynamics between individuals, the adaptive value of engaging in social interactions (Höjesjö, Johnsson & Bohlin, 2004; Banks *et al*., 2007; Tanner & Jackson, 2012; Fisher *et al*., 2021; Blumstein *et al*., 2023), or the physiological responses that mediate social behaviour (Seebacher & Krause, 2017; Batabyal & Thaker, 2019; Mills *et al*., 2020). Given the drastic changes in biotic and abiotic conditions caused by urbanisation, animals living in urban habitats are likely to face different selection pressures on social behaviour relative to non‐urban populations. Like other traits, animals should optimise their social behaviour through plastic or evolved responses for an optimal individual–environment match (Wright *et al*., 2010; Miranda, 2017). There is growing evidence of the impact urbanisation is having on animal social systems, with individual‐, group‐, and population‐level responses.

Previous reviews have examined how urbanisation may influence social behaviour by focusing on a specific urban stressor, taxon, or behaviour, or by discussing anthropogenic environmental change more broadly. For example, previous work has discussed how anthropogenic noise affects social behaviour by analysing the effect of noise on acoustic communication (Brumm & Slabbekoorn, 2005; Warren *et al*., 2006; Kunc & Schmidt, 2021), and by focusing on how anthropogenic noise affects specific taxa [fish (Radford, Kerridge & Simpson, 2014); birds and amphibians (Roca *et al*., 2016); mammals (Bednarz, 2021); insects and arachnids (Classen‐Rodríguez, Tinghitella & Fowler‐Finn, 2021)]. Kurvers & Hölker (2015) discussed how artificial light at night (ALAN) is expected to affect social structure. Regarding mating systems, Cronin *et al*. (2022b) outlined how urbanisation shapes sexual signalling and sexual selection; Heinen‐Kay, Kay & Zuk (2021) reviewed how urbanisation impacts sexual communication; and Candolin (2019) discussed how human‐induced environmental change is affecting mate choice. Focusing on environmental change more broadly, Blumstein *et al*. (2023) outlined a framework for how environmental change impacts social behaviour and structure, while Fisher *et al*. (2021) reviewed how abiotic environmental change affects intraspecific social interactions. However, we are lacking a comprehensive review on how urbanisation, as a distinct form of environmental change, is influencing social behaviour.

Here, we aim to fill this gap by providing the first review addressing the impact of urbanisation on social behaviour and suggesting a framework for future research. Specifically, we (*i*) describe how different biotic and abiotic stressors inherent to urbanisation (i.e. urban stressors) can affect social behaviour, (*ii*) discuss how social systems can differ between urban and non‐urban populations, (*iii*) describe the impact of urbanisation on interspecific social interactions, (*iv*) synthesise general trends and anticipated social responses, and (*v*) highlight biases and gaps in the current knowledge and suggest avenues for future research. While we primarily focus on studies from our systematic literature search, we also discuss additional research that addressed the impact of environmental change on social behaviour, to highlight how urbanisation is predicted to affect social behaviour in areas that have received less research attention. It is important to note that urban habitats vary substantially, not only in structure and human population density, but also in the type and intensity of urban stressors, the degree of habitat fragmentation, and in connectivity to surrounding natural areas (Cadenasso, Pickett & Schwarz, 2007). What constitutes an urban area can differ markedly across regions and cultures, with definitions of cities and urban infrastructure varying worldwide (United Nations, Department of Economic and Social Affairs, Population Division, 2019). We therefore do not impose a strict definition of urbanisation, but instead rely on the classifications used by authors, while acknowledging that these differences may underlie some of the variation in social responses discussed throughout this review. This review represents an important step in understanding how urbanisation impacts animal social systems, which is imperative to addressing species responses to human‐induced rapid environmental change.

## II. LITERATURE SEARCH

**Fig. 1:**
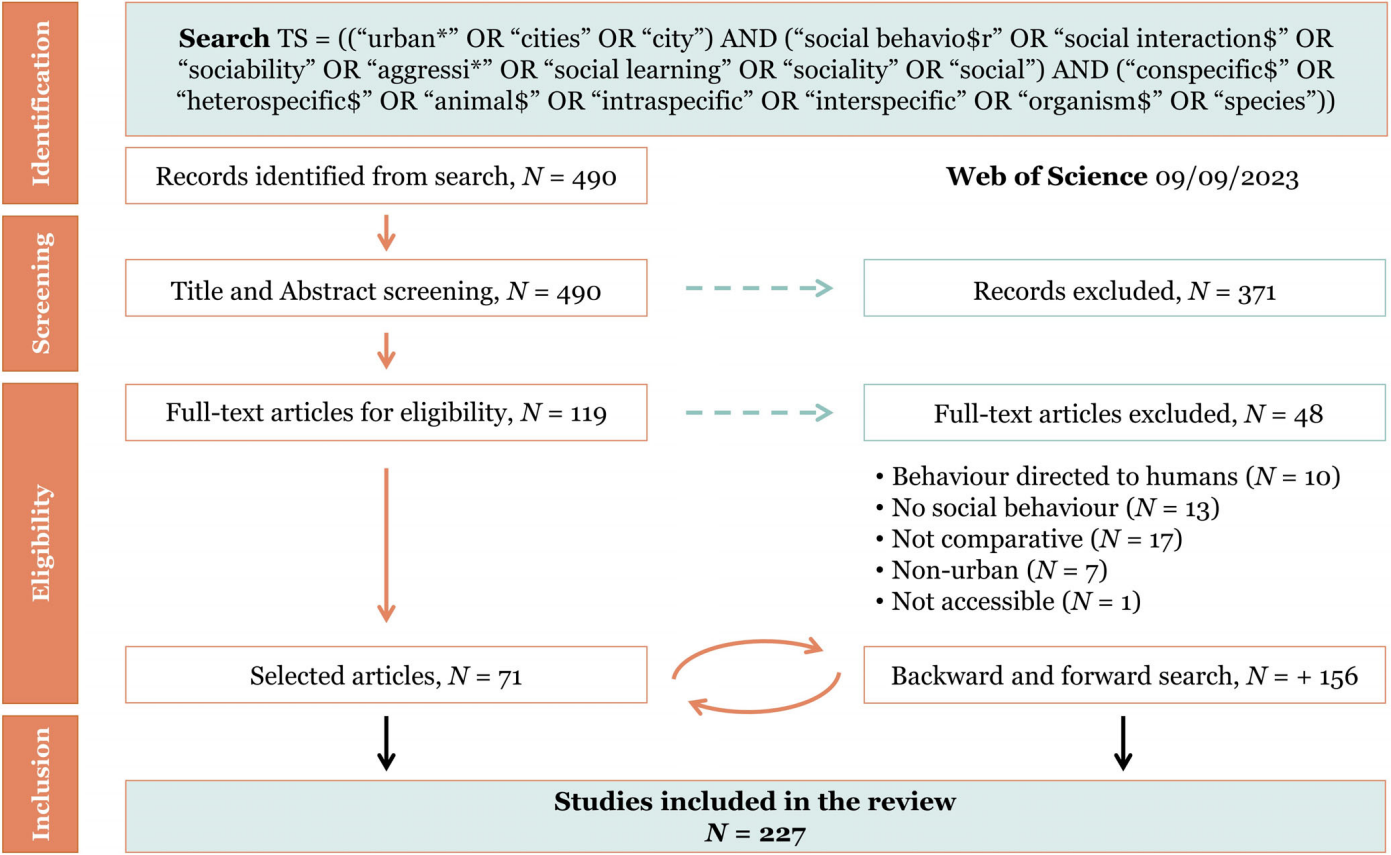
PRISMA flow chart of the systematic literature search, which resulted in a total of 227 papers.

We conducted a search in *Web of Science* within the Citation Topics Meso 3.35 Zoology & Animal Ecology using the following search terms: TOPIC: ‘urban*’ or ‘cities’ or ‘city’ AND ‘social behavio$r’ or ‘social interaction$’ or ‘sociability’ or ‘aggressi*’ or ‘social learning’ or ‘sociality’ or ‘social’ AND ‘conspecific$’ OR ‘heterospecific$’ OR ‘animal$’ OR ‘intraspecific’ OR ‘interspecific’ OR ‘organism$’ OR ‘species’ (search summarised in Fig. 1). We performed the search in September 2023 and retrieved 490 records, which were screened based on title and abstract to assess relevance. This yielded 119 papers that were retained for full‐text screening. From these, we selected original research papers that either (*i*) experimentally tested the effect of an urban stressor on social behaviour, (*ii*) compared social behaviour between populations experiencing different levels of exposure to an urban stressor, (*iii*) compared changes in social behaviour within a population that experienced changing conditions over time related to urbanisation, or (*iv*) compared social behaviour between urban and non‐urban populations. We did not define what constitutes an urban habitat and instead relied on the authors' specification. We excluded (*i*) reviews and meta‐analyses, (*ii*) studies on domestic animals, (*iii*) studies that either were not performed in urban settings or did not test the effect of an urban stressor, (*iv*) studies that measured behaviour directed towards humans, and (*v*) heterospecific studies that do not describe a behaviour directed towards another individual (e.g. exploitative competition, space‐use patterns, or predation). This resulted in 71 papers. From these selected papers, we then performed a backward and forward literature search of their citations and retrieved an additional 156 papers. The difference between the final and initial search reflects the fact that the majority of the papers from the backward–forward search (79%) were investigating the impact of anthropogenic noise on communication, which is often framed in relation to anthropogenic change (i.e. words related to urbanisation were not present in the title, abstract, or key words), and thus did not appear in our initial search. A total of 227 papers were included in our review, with 218 addressing intraspecific social behaviour, and 12 addressing interspecific social behaviour (three papers addressed both). To assess the completeness of our search, we repeated our search in *Scopus* for five representative years (2006, 2011, 2017, 2019, 2022), including backward and forward citation searches of relevant papers. Across these years, we identified only three papers that were not captured through our original search methods (96% recall), which were not included in our final data set. Thus, we are confident that our extensive literature search was thorough and captured the majority of relevant studies.

## III. EFFECTS OF URBAN STRESSORS ON SOCIAL BEHAVIOUR

Animals living in urban areas must contend with a range of novel and interacting stressors, including human activity, anthropogenic food, urban structures, anthropogenic noise, ALAN, and chemical pollutants. Below, we describe how each of these urban stressors can impact social behaviour (see Table 1, for summary of included studies and outcomes). We provide empirical examples from our literature search and, where necessary, include additional examples from areas where research is lacking.

### (1) Human activity

Animals inhabiting urban areas must cope with the near‐constant presence of humans, which can lead to complicated relationships between humans and wildlife. While positive interactions, such as food provisioning, may lead animals to habituate to, or seek out, humans, competition for resources and space can escalate conflict between humans and urban animals (Basak *et al*., 2023), and the nature of these interactions can influence intraspecific social behaviour. For example, if humans are perceived as a threat, heightened vigilance can lead to behavioural modifications that disrupt social interactions, as observed in California ground squirrels (*Otospermophilus beecheyi*), which reduced social foraging and affiliative interactions in response to disturbances from humans and dogs (Gall *et al*., 2022). Because animals have a limited amount of time and energy to invest in daily activities, individuals focused on monitoring human activity will have less time available for interacting with conspecifics. Rhesus and bonnet macaques (*Macaca mulatta* and *Macaca radiata*, respectively) living in areas with high human activity spent more time monitoring humans and had shorter and less‐frequently reciprocated grooming bouts (Kaburu *et al*., 2019a,b; Balasubramaniam *et al*., 2020). This time constraint can disrupt social bonds and reduce cohesion within groups, which may be particularly detrimental for species with complex social systems.

Alternatively, social interactions can act as a coping mechanism for heightened stress associated with human presence. The ‘social buffering hypothesis’ suggests that affiliative and prosocial behaviours can reduce physiological responses to stress (Culbert, Gilmour & Balshine, 2019; Wu, 2021). Given that human presence has been linked to heightened physiological stress responses in several taxa [e.g. birds (Müllner, Linsenmair & Wikelski, 2004); reptiles (French *et al*., 2010); mammals (Fourie *et al*., 2015)], some urban animals may invest more time and energy in social relationships to cope with human activity, which in turn has the potential to strengthen social cohesion within groups. This was observed in mountain gorillas (*Gorilla beringei*), which performed more prosocial behaviours and spent more time in close association with group members when tourists were nearby (Costa *et al*., 2023a,b).

Behavioural responses to humans can depend on the intensity of human activity and the nature and predictability of human behaviour. For example, long‐tailed macaques (*Macaca fasicularis*) living in sites with moderate human activity were observed to spend more time monitoring humans at the cost of reduced grooming behaviour, while those at sites with higher levels of human activity exhibited a heightened stress response which was positively associated with social interactions (Marty *et al*., 2019). Positive interactions with humans (e.g. receiving food provisions) led to increased social interactions in urban vervet monkeys (*Chlorocebus pygerythrus*), while negative social interactions (e.g. receiving aggression) diminished social behaviour (Thatcher, Downs & Koyama, 2019). Rhesus macaques (*Macaca mulatta*) who experienced equal rates of positive and negative human interactions spent more time monitoring humans to understand their intentions, leading to reduced grooming behaviour (Kaburu *et al*., 2019a).

Human presence can, directly or indirectly, impact social interactions and group functioning in multiple, and sometimes opposing, ways. The direction of these effects can be highly variable and species or context dependent. All the studies described above were conducted on species that form social groups or exhibit more complex social behaviours, where high levels of human activity can reduce the time available for social interactions, leading to weakened social bonds and reduced group cohesion. Although some species may use social interactions as a coping mechanism to buffer against human‐induced stress, this has not been well addressed in urban environments, where exposure to humans is more pervasive. Given that human presence is a ubiquitous feature of cities, it is likely also affecting ‘less‐social’ species (e.g. French *et al*., 2018), yet this has rarely been addressed. Future research should explore how social flexibility, risk sensitivity, and life‐history traits mediate responses to human activity across a broader range of taxa. Importantly, social responses to humans may vary depending on local context, such as the degree of human–wildlife conflict and the attitudes of urban residents towards wildlife.

### (2) Anthropogenic food

Urban animals often exploit anthropogenic food sources, provided either inadvertently (e.g. pet food, garbage) or deliberately (e.g. bird feeders, human provisions) by humans, which can offer a predictable and easily accessible source of energy (Fehlmann *et al*., 2021). Anthropogenic food is generally temporally and spatially predictable (Oro *et al*., 2013), which may alleviate competition and aggression in urban environments. In line with this, the ‘credit‐card hypothesis’ predicts that urban habitats may be able to support weaker competitors if access to food is constant (Shochat, 2004). Hasegawa *et al*. (2014) tested this experimentally in house finches (*Haemorhous mexicanus*) and found that birds from urban populations were indeed less competitive. Conversely, consistent access to anthropogenic food may allow animals more time and energy to invest in territorial defence or social interactions. Interestingly, urban and non‐urban song sparrows (*Melospiza melodia*) receiving supplemental food increased their territorial aggression, particularly in non‐urban populations, suggesting that resource abundance may be the limiting factor driving differences in aggression observed between some urban and non‐urban bird populations (Foltz *et al*., 2015). Moreover, if anthropogenic food is perceived as higher quality, urban individuals may be more motivated to defend those resources aggressively (Theimer *et al*., 2015; Fountain & McDonald, 2022). A study comparing mesocarnivore responses to cat food and bird seed, two common anthropogenic food sources, found that cat food, which is likely perceived as higher quality, attracted significantly more individuals and resulted in more aggressive interactions between striped skunks (*Mephitis mephitis*) (Theimer *et al*., 2015). However, consistent access to food can also allow individuals to reduce their time spent foraging, freeing up opportunities to invest in social relationships (Jaman & Huffman, 2013), which can strengthen social bonds and increase within‐group cohesion.

The abundance and distribution of anthropogenic resources in the environment can also significantly affect social behaviour and mediate group size (Carr & MacDonald, 1986; Johnson *et al*., 2002; Newsome *et al*., 2013). For example, if anthropogenic food sources are clustered together, individuals may aggregate in higher numbers, potentially leading to intensified competition and aggression (Flint, Hawley & Alexander, 2016). Mareeba rock‐wallabies (*Petrogale masseri*) and African lesser bushbabies (*Galago moholi*) were observed to aggregate around food provisioned by tourists, resulting in increased intraspecific aggression (Hodgson, Marsh & Corkeron, 2004; Scheun *et al*., 2015). Interestingly, however, urban bushbabies also exhibited a higher‐than‐expected level of cohesion between conspecifics and formed social groups (Scheun, Greeff & Nowack, 2019). Similarly, alpine choughs (*Pyrrhocorax graculus*) formed larger groups when foraging in tourist areas with access to anthropogenic food (Jiménez *et al*., 2013). Clumped resources have also been shown to promote sociality in other species (Tanner & Jackson, 2012), and higher resource abundance may support larger group sizes (Newsome *et al*., 2013).

Whether anthropogenic food reduces competition and promotes tolerance, or instead intensifies aggression and social instability, likely depends on its predictability and spatial distribution, as well as a species' innate social tendencies. Strictly territorial species may respond with heightened aggression, while others may relax territorial boundaries under greater resource abundance or clumped distributions. While anthropogenic food is likely to promote larger aggregations and group sizes overall, this may not necessarily translate into stronger social bonds or tolerance (Belton, Cameron & Dalerum, 2018). Variation in responses may be shaped by a species' social structure, dietary niche, or foraging strategies, and future work should explore how these traits mediate behavioural responses to anthropogenic food in urban environments. Studies in natural settings investigating how different resource distributions influence social structure and grouping patterns can help shed light on how anthropogenic food in cities is influencing social behaviour.

### (3) Urban structures

Urban landscapes are highly structured and have markedly different habitat configurations from natural areas (Grimm *et al*., 2008). Buildings and roads can restrict the movement and dispersal ability of animals (Fahrig, 2007; Taylor & Goldingay, 2010), which can in turn increase local population densities and influence social structure. An experimental manipulation of habitat complexity in sleepy lizards (*Tiliqua rugosa*) found that when the habitat was more structured, changes in movement patterns increased the encounter rate among individuals and led to more social interactions than usual for this species (Leu *et al*., 2016). Similarly, a study tracking the movement patterns of coachwhip snakes (*Masticophis flagellum*) found that reduced movement in fragmented habitats resulted in increased crowding and territory overlap (Mitrovich, Diffendorfer & Fisher, 2009). More frequent social encounters can lead to heightened aggression, or alternatively, may promote social tolerance or the formation of dominance hierarchies, which can reduce conflict and competition at higher densities. For group‐living species, structural barriers in cities may impede collective movement and isolate group members, leading to the breakdown of social groups in urban areas (Banks *et al*., 2007; Bracken *et al*., 2022).

Human‐made structures can significantly alter the propagation and transmission of visual and acoustic signals, which can have obvious impacts on social interactions between senders and receivers. For example, effective visual communication is dependent on a sufficient line of sight between the sender and the receiver, and reduced vegetation in urban habitats can enhance the visibility of conspecifics, leading to more frequent social encounters. Cuban brown anoles (*Anolis sagrei*) living in urban habitats with reduced understory were observed to perform significantly more visual displays compared to individuals living in forested habitats (Stroud *et al*., 2019). As visual displays play an important role in mediating conflict between conspecifics, enhanced visibility may decrease the need to escalate aggressive behaviour (Baird, Baird & Shine, 2020). However, urban structures can impact acoustic communication in a very different way because, unlike vegetation, which absorbs some sound energy, impervious surfaces are highly reflective, causing acoustic signals to reverberate and become distorted (Warren *et al*., 2006). To overcome this, animals can lower the frequency of acoustic signals to reduce reverberation, or temporally separate syllables, making them clearer and easier to detect (Potvin, Parris & Mulder, 2011; Hill *et al*., 2018). Acoustic signals that evolved in structurally complex environments, such as forests, may transmit more effectively in urban habitats than signals that evolved in open habitats. For example, song features such as trills and high frequencies transmit well in open habitats but are more likely to degrade in urban environments, where dense vertical structures can resemble forested habitats (Gall *et al*., 2012; Job, Kohler & Gill, 2016). However, urban areas are highly variable in their structure, and the extent to which a given signal is effective will depend on the specific habitat features, such as the height and density of buildings.

Impermeable surfaces have a high heat retention capacity, which can lead to a phenomenon known as ‘urban heat islands’ where urban habitats have significantly higher temperatures than surrounding natural areas (Phelan *et al*., 2015). While our search did not identify any studies on this, temperature is known to impact social behaviour directly and indirectly (reviewed by Moss & While, 2021). For example, changes in the thermal environment can fundamentally impact social systems by mediating the energy available for engaging in social interactions or investing in reproductive behaviours (Fisher *et al*., 2021). Furthermore, for species with temperature‐dependent sex determination, higher temperatures in cities may lead to altered sex ratios, impacting sex‐specific interactions (Mitchell & Janzen, 2010; Blumstein *et al*., 2023). High temperatures may also impede chemical communication by decreasing the efficacy of chemical cues (Martín & López, 2013; Iglesias‐Carrasco *et al*., 2018).

Overall, the extent and nature of social responses to urban infrastructure is likely to be largely dependent on the spatial configuration of the urban matrix, as well as species' movement ecology. Species with greater dispersal abilities (e.g. birds) may be better able to navigate fragmented landscapes and maintain social connections across space, while species with limited mobility will likely experience more pronounced changes in encounter rates or group cohesion. These effects may be compounded by disrupted communication due to impaired signal transmission or physiological responses to elevated temperatures within cities. Future work integrating tracking technologies with social network approaches offers a powerful approach to quantify how urban infrastructure influences collective movement, encounter rates, and group dynamics (e.g. Bracken *et al*., 2022).

### (4) Anthropogenic noise

Anthropogenic noise is a pervasive consequence of urbanisation that can have profound effects on behaviour across taxa. The impact of anthropogenic noise on social behaviour has been relatively well studied, with considerable research focusing on how it affects acoustic communication (Brumm & Slabbekoorn, 2005). High amplitude, low‐frequency sounds from traffic, construction, aircrafts, and boats (Can *et al*., 2010) can overlap with the acoustic signals of terrestrial and marine species, and disrupt the transmission and detection of signals (Vasconcelos, Amorim & Ladich, 2007; Pohl *et al*., 2009; Kern & Radford, 2016; Templeton, Zollinger & Brumm, 2016; Nelson *et al*., 2017; Grabarczyk & Gill, 2020). Given that effective communication is crucial for mediating conflict, maintaining social relationships, selecting mates, and coordinating collective behaviour, exposure to anthropogenic noise can have substantial downstream effects on social behaviour. For example, anthropogenic noise can affect territorial behaviour by interfering with signals used to mediate conflict, or by impairing the territory holder's ability to deter or assess accurately the threat posed by an intruder, potentially causing the territory holder to escalate to higher levels of aggression (Phillips & Derryberry, 2018; Akçay *et al*., 2020b; Önsal, Yelimlieş & Akçay, 2022). Male house wrens (*Troglodytes aedon*) and European robins (*Erithacus rubecula*) respond more aggressively to simulated territorial intrusions in the presence of anthropogenic noise, suggesting that noise may play a causal role in the heightened aggression observed in some urban populations (Grabarczyk & Gill, 2019a; Önsal *et al*., 2022). Conversely, territory holders may instead fail to respond appropriately to intruders and act less aggressively when exposed to anthropogenic noise (Sebastianutto *et al*., 2011; Kleist *et al*., 2016; Zwart *et al*., 2016; Passos *et al*., 2020), which can impair their ability to hold territories, to the detriment of reproductive success.

Similarly, anthropogenic noise can alter within‐ and between‐group dynamics. Exposure to traffic noise impaired communication and resulted in reduced between‐group interactions in fairy wrens (*Malurus melanocephalus*) (Hawkins, Ritrovato & Swaddle, 2020). Conversely, Carolina chickadees (*Poecile carolinensis*) and tufted titmice (*Baeolophus bicolor*) reduced their nearest‐neighbour distance, which was hypothesised to be an anti‐predator response to traffic noise (Owens, Stec & O'Hatnick, 2012). Exposure to noise increased the amount of aggression received and submission shown by subordinate cichlid fish (*Neolamprologus pulcher*) (Bruintjes & Radford, 2013). A subsequent study found that females, but not males, exhibited more submissive displays, yet were less affiliative, following exposure to noise (Braga Goncalves *et al*., 2021). By impairing effective communication, anthropogenic noise can also disrupt the coordination of collective and cooperative behaviours. Shoaling seabass (*Dicentrarchus labrax*) exposed to anthropogenic noise were less cohesive and less effective in coordinating group movements (Herbert‐Read *et al*., 2017), while bottlenose dolphins (*Tursiops truncatus*) were unable to coordinate their behaviour to solve a cooperative task effectively (Sørensen *et al*., 2023).

Many species use acoustic signals to attract and assess mates, which can be masked by anthropogenic noise (Bee & Swanson, 2007; Halfwerk, Bot & Slabbekoorn, 2012; Vieira *et al*., 2021). Sexual signals that are adjusted to overcome masking may conflict with established mate preferences, potentially disrupting mating behaviours or shifting patterns of sexual selection (Halfwerk *et al*., 2011; Huet des Aunay *et al*., 2014). Anthropogenic noise may also impair parent–offspring communication and disrupt the coordination of care behaviours between parents (Mariette, 2019). Thus, anthropogenic noise can have direct effects on reproductive success.

To enhance signal transmission in noisy conditions, animals can alter the structure, timing, or effort of their vocalisations. For example, birds often shift the frequency of their songs in response to anthropogenic noise (e.g. Slabbekoorn & Peet, 2003; Wood & Yezerinac, 2009; Bermúdez‐Cuamatzin *et al*., 2009, 2010; Verzijden *et al*., 2010; Proppe *et al*., 2012; Luther & Derryberry, 2012; Redondo, Barrantes & Sandoval, 2013; Luther, Phillips & Derryberry, 2016; Warrington *et al*., 2018; Walters *et al*., 2019; Sheldon *et al*., 2020; Zhan *et al*., 2021), which can improve signal detection (Pohl *et al*., 2012; LaZerte, Otter & Slabbekoorn, 2017a), and has also been observed in insect (Lampe *et al*., 2012) and anuran species (Parris, Velik‐Lord & North, 2009; Kruger & Du Preez, 2016; Leon *et al*., 2019; Grenat *et al*., 2019; Jiménez Vargas & Vargas Salinas, 2021; Higham *et al*., 2021; Zhao *et al*., 2021), although not as commonly. Animals can also increase the amplitude of their acoustic signals (i.e. the ‘Lombard effect’, Brumm & Zollinger, 2011) which has been observed in a variety of taxa, such as birds (Lowry, Lill & Wong, 2012; Nemeth *et al*., 2013; Potvin & Mulder, 2013; Zollinger *et al*., 2017), amphibians (Yeo & Sheridan, 2019; Leon *et al*., 2019; Lima *et al*., 2022), and mammals (Parks *et al*., 2010; Jiang *et al*., 2019; Sørensen *et al*., 2023). Alternatively, some species exhibit temporal changes in vocal activity, shifting the duration (Halfwerk & Slabbekoorn, 2009; McMullen, Schmidt & Kunc, 2014; Potvin & MacDougall‐Shackleton, 2015; Ritz‐Radlinská *et al*., 2023), rate (Sun & Narins, 2005; Ríos‐Chelén *et al*., 2013; Ríos‐Chelén, Lee & Patricelli, 2015; Kruger & Du Preez, 2016; Injaian, Lane & Klinck, 2021; Jézéquel, Bonnel & Chauvaud, 2021; Önsal *et al*., 2022; Sánchez, Bayne & Hilje, 2023), or timing (Fuller, Warren & Gaston, 2007; Cartwright *et al*., 2014; Bermúdez‐Cuamatzin *et al*., 2020; Sathyan & Couldridge, 2021; Gallardo Cruz, Paxton & Hart, 2021; de Framond & Brumm, 2022) of vocal activity to minimise interference from anthropogenic noise and enhance signal transmission.

There is substantial variability in how animals adjust acoustic signalling in response to anthropogenic noise (Roca *et al*., 2016; Kunc & Schmidt, 2021), which can be influenced by the intensity of noise (Caorsi *et al*., 2017; Yeo & Sheridan, 2019), innate vocal characteristics (Hu & Cardoso, 2010; Zhao *et al*., 2021), or the social environment (Lengagne, 2008; Higham *et al*., 2021). Some animals exhibit immediate flexibility in response to anthropogenic noise [birds (Bermúdez‐Cuamatzin *et al*., 2010; Verzijden *et al*., 2010; Hanna *et al*., 2011); anurans (Cunnington & Fahrig, 2010; Zhao *et al*., 2021; Grenat *et al*., 2023)], which may be particularly beneficial in urban environments with fluctuating noise levels, and influenced by prior experience (LaZerte, Slabbekoorn & Otter, 2016, 2017a; Gentry *et al*., 2017) or dependent on whether vocalisations are innate or learned (Ríos‐Chelén *et al*., 2012; Rivera‐Gutierrez *et al*., 2023).

To compensate for disruptions to acoustic signalling, animals can switch to, or increase the rate of, other signalling modalities, such as visual or olfactory cues (Brumm & Slabbekoorn, 2005). Multi‐modal signals act redundantly, increasing the likelihood of signal detection in case of failure in one modality (Akçay & Beecher, 2019). Partan *et al*. (2010) presented grey squirrels (*Sciurus carolinensis*) with a robotic squirrel that produced visual and acoustic alarm signals and found that squirrels responded more strongly when both modalities were present. Additionally, urban squirrels responded more strongly to visual displays than non‐urban squirrels, suggesting a modal shift in communication. Evidence of a multi‐modal shift in response to noise has also been observed in pied tamarins (*Saguinus bicolor*) (Sobroza *et al*., 2023) and painted gobies (*Pomatoschistus pictus*) (de Jong *et al*., 2018b).

Anthropogenic noise can further impact social behaviour through physiological responses or impaired cognitive abilities. Exposure to noise can increase stress levels (Kight & Swaddle, 2011), which can subsequently alter communication signals, such as colouration (Troïanowski *et al*., 2015), or lead to heightened aggression (Mills *et al*., 2020). Anthropogenic noise can also interfere with the ability to process information and make adaptively appropriate decisions (i.e. cross‐modal interference), which can disrupt species' typical patterns of behaviour (Chan & Blumstein, 2011; Halfwerk & Slabbekoorn, 2015). For example, noise reversed natural grouping patterns in hermit crabs (*Pagurus bernhardus*), causing crabs in suboptimal shells to behave as though they had higher quality shells and group with conspecifics (Tidau & Briffa, 2019).

While there is a large body of research on how noise affects acoustic communication, this has largely focused on birds and anurans, and there is a need for research on a broader range of taxa. Moreover, species vary in their sensitivity and behavioural flexibility in response to anthropogenic noise, and this variation may be shaped by traits such as vocal plasticity, reliance on acoustic communication, the modality of signalling, and prior exposure to noisy environments. We still know relatively little about the downstream social consequences of these disruptions. Future research should strive to evaluate the response of both signal senders and receivers to understand fully how noise interferes with communication dynamics. Further research is also needed to understand how the cognitive and physiological impacts of chronic noise exposure contribute to changes in social behaviour and interaction patterns.

### (5) Artificial light at night

ALAN can disrupt natural light–dark cycles that mediate circadian rhythms and activity patterns, which can have significant consequences for social behaviour (for a review of predicted impacts see Kurvers & Hölker, 2015). For example, exposure to ALAN can affect photoperiods, which many species rely on to synchronise and time reproductive behaviours, including courtship, mating, and parental care (Gaston *et al*., 2013). There is evidence that bird species breeding near artificial light advance the date of egg‐laying (Kempenaers *et al*., 2010; Dominoni, Quetting & Partecke, 2013), start singing their dawn chorus earlier (Miller, 2006; Kempenaers *et al*., 2010), and increase provisioning rates (Titulaer *et al*., 2012). These shifts may lead to temporal mismatches between social partners, potentially disrupting mating behaviours or parental care, and undermining the effectiveness of social interactions critical for reproductive success.

Exposure to ALAN can also lead to maladaptive behaviours that inhibit reproductive success and is likely particularly impactful for nocturnal breeding species or species that use light in courtship behaviours. Female common glow‐worms (*Lampyris noctiluca*) exposed to artificial lights failed to move away, and delayed, or avoided, glowing, and consequently attracted fewer males (Elgert *et al*., 2020). Male green frogs (*Rana clamitans*) reduced their calling behaviour when exposed to artificial light to avoid predator detection (Baker & Richardson, 2006). Exposure to bright light weakened mate discrimination in female field crickets (*Teleogryllus commodus*), while males reared in bright light experienced more discrimination (Botha, Jones & Hopkins, 2017).

ALAN may also increase the rate of social interactions through heightened visibility of conspecifics and conspicuousness of visual signals (Kurvers & Hölker, 2015). For example, mammals often exhibit lower levels of aggression at night, which has been attributed to decreased visibility of cues that trigger aggressive behaviour (Beauchamp, 2007). ALAN may therefore increase aggressive interactions in some species. Additionally, light can attract animals, particularly insects, which in turn may attract nocturnal species that consume insects, such as bats or geckos (Minnaar *et al*., 2015), potentially leading animals to aggregate around artificial light sources, and increasing the opportunity for social encounters (Kurvers & Hölker, 2015).

Despite the impact that exposure to ALAN is likely having on important reproductive and social behaviours, its effects on social behaviour remain poorly understood. Future studies should investigate how ALAN influences the temporal coordination of social behaviours that rely on sensory cues and reproductive timing, as well as how physiological and circadian mechanisms mediate these effects. Experimental approaches that manipulate light exposure can be valuable and easily implemented for identifying thresholds at which ALAN begins to interfere with social behaviours, and whether these responses are reversible.

### (6) Chemical pollutants

Chemical pollutants can significantly impact health and physiology, disrupting endocrine systems and neurological functioning, which ultimately affect behaviour (for reviews see Blocker & Ophir, 2013; Gore, Krishnan & Reilly, 2019; Michelangeli *et al*., 2022). Pollutants can act on neural signalling and transmission or endocrine systems, leading to behavioural modifications, such as changes in aggression (Bell, 2001) and mating behaviour (Secondi *et al*., 2009; Whitlock *et al*., 2018), which can differ according to social status (McCallum *et al*., 2021). Furthermore, reduced health due to chemical pollutants can limit the energy available for social interactions. Urban colonies of the ant *Temnothorax nylanderi* exhibit reduced aggression towards intruders, which was hypothesised to be due to metal pollutants negatively affecting the health of worker ants (Jacquier, Molet & Doums, 2023). Conversely, urban mockingbirds (*Mimus polyglottos*) living in areas with high lead soil concentrations exhibited more territorial aggression compared to those living in areas with low lead soil concentrations (McClelland *et al*., 2019). By impacting health and physiological processes, exposure to chemical pollutants can also affect communication and sexual signals, such as colouration (Chatelain *et al*., 2017; Grunst *et al*., 2020), olfactory cues (Lürling & Scheffer, 2007; Secondi *et al*., 2009; Blocker & Ophir, 2013), or acoustic signals (Gorissen *et al*., 2005; Whitlock *et al*., 2018). Lethal exposure to chemical pollutants can change patterns of social organisation and alter mating systems by limiting the number of individuals available to interact or mate with, or by altering phenotypic variation within social groups (Michelangeli *et al*., 2022).

There is substantial evidence that chemical pollutants can impact social behaviour. However, there is a need for studies that quantify the composition and distribution of chemical pollutants within urban habitats and directly link these to behavioural and physiological outcomes. Pollutants can induce subtle or inconspicuous physiological changes that manifest behaviourally, but these effects may be overlooked or attributed to other urban stressors. Future research should aim to disentangle the specific contribution of chemical pollution to social systems in urban environments. Experimental and longitudinal studies that assess dose‐dependent effects or multigenerational consequences can be particularly valuable for understanding how severe and persistent these changes may be.

**Table 1:** Summary of outcomes from the 227 papers retrieved through our literature search. ‘Urban outcome’ refers to the results found for the social measure investigated in the studies (0 indicates no urban impact was found, while + and – represent increasing and decreasing trends, respectively). ALAN refers to artificial light at night. Note that this table provides a simplified summary to identify trends in relevant papers, and the studies listed may contain more nuance and detail than outlined in this table. Papers that examine multiple behaviours or aspects of urbanisation may appear in the table more than once.

Social system	Urban topic	Social measure		Taxa	Urban outcome		References
**Social organisation**	**Urban *vs* non‐urban**	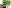	group size	Mammals	**+**	larger group size	Scheun *et al*. (2019)
					**−**	smaller group size	Bracken *et al*. (2022)
				Birds	**−**	smaller non‐breeding groups	Kucera *et al*. (2019)
					**0**	no difference in group size	Beck & Heinsohn (2006)
				Reptiles	**+**	larger group size	Lacy & Martins (2003)
			group composition	Birds	**0**	no difference in group composition	Beck & Heinsohn (2006)
			group cohesion	Mammals	**+**	more cohesive	Scheun *et al*. (2015)
					**−**	less cohesive	Bracken *et al*. (2022)
			connectivity	Birds	**0**	no difference between social networks	Jones *et al*. (2019)
	**Human activity**	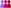	group size	Birds	**−**	smaller group size	Jiménez *et al*. (2013)
			group cohesion	Mammals	**+**	increased cohesion	Costa *et al*. (2023a)
					**−**	reduced cohesion	Morrow *et al*. (2019)
			connectivity	Mammals	**−**	reduced connectivity	Belton *et al*. (2018); Morrow *et al*. (2019); Gall *et al*. (2022)
	**Anthropogenic food**	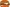	group size	Birds	**+**	increased group size	Jiménez *et al*. (2013)
	**Anthropogenic noise**	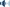	group size	Crustacea	**+/−**	increased or decreased according to individual traits	Tidau & Briffa (2019)
			group cohesion	Fish	**−**	reduced cohesion	Herbert‐Read *et al*. (2017)
**Social structure**	**Urban *vs* non‐urban**	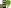	collective behaviour	Mammals	**−**	disrupted collective movement	Bracken *et al*. (2022)
			social information	Birds	**−**	less reliant on social information	Jones *et al*. (2017); Morand‐Ferron *et al*. (2019)
			rate of social interactions	Mammals	**+**	higher rate of grooming behaviour	Jaman & Huffman (2013); Scheun *et al*. (2015)
					**0**	no difference in time socialising	Khatun *et al*. (2018)
				Birds	**+**	higher rate of social interactions	Price & Hayes (2017)
			aggression	Mammals	**+**	more aggressive	Scheun *et al*. (2015)
					**−**	less aggressive	Łopucki *et al*. (2021)
					**0**	no difference in aggression	Hurtado & Mabry (2017); Uchida *et al*. (2020)
				Birds	**+**	more aggressive	Evans *et al*. (2010); Ripmeester *et al*. (2010); Scales *et al*. (2011); Fokidis *et al*. (2011); Foltz *et al*. (2015); Davies & Sewall (2016); Myers & Hyman (2016); Hardman & Dalesman (2018); LaZerte *et al*. (2019); Akçay *et al*. (2020a); Diniz & Duca (2021); Lane & Sewall (2022); Önsal *et al*. (2022)
					**−**	less aggressive	Hasegawa *et al*. (2014, 2018)
				Reptiles	**+**	more aggressive	Lacy & Martins (2003); Baxter‐Gilbert & Whiting (2019)
					**−**	less aggressive	Batabyal & Thaker (2019)
				Insects	**−**	less aggressive	Jacquier *et al*. (2023)
				Arachnids	**−**	less aggressive	Kralj‐Fišer *et al*. (2017); Daniel & Chaves‐Campos (2021)
			visual signals	Reptiles	**+**	higher display rate	Stroud *et al*. (2019)
					**−**	fewer displays or slower colour change	Batabyal & Thaker (2017, 2019); Vidal *et al*. (2023)
			acoustic signals	Mammals	**+**	higher frequency	Starik & Göttert (2022)
				Birds	**+**	higher frequency or amplitude	Slabbekoorn *et al*. (2007); Mockford & Marshall (2009); Nemeth & Brumm (2009); Hu & Cardoso (2009, 2010); Ripmeester *et al*. (2010); Potvin *et al*. (2011); Dowling *et al*. (2012); Nemeth *et al*. (2013); Redondo *et al*. (2013); Narango & Rodewald (2016); LaZerte *et al*. (2017a); Lowry *et al*. (2019); Phillips *et al*. (2020b); Reichard *et al*. (2020); Diniz & Duca (2021); Cyr *et al*. (2021)
					**+**	longer duration or higher rate	Nemeth & Brumm (2009); Redondo *et al*. (2013); Narango & Rodewald (2016); Hill *et al*. (2018); Phillips *et al*. (2020b); Cyr *et al*. (2021)
					**−**	lower frequency	Potvin *et al*. (2014); Brewer & Fudickar (2022); Yelimlieş *et al*. (2023)
					**−**	shorter duration or lower rate	Potvin *et al*. (2011); Hill *et al*. (2018); Phillips *et al*. (2020b); Cyr *et al*. (2021); Önsal *et al*. (2022)
					**0**	no difference in frequency	Hu & Cardoso (2009, 2010); Dowling *et al*. (2012); Derryberry *et al*. (2016); Lowry *et al*. (2019); Smith‐Vidaurre *et al*. (2021); Deoniziak & Osiejuk (2021); Yelimlieş *et al*. (2023)
					**0**	no difference in duration or rate	Slabbekoorn *et al*. (2007); Mockford & Marshall (2009); Lowry *et al*. (2019); Deoniziak & Osiejuk (2021); Brewer & Fudickar (2022); Önsal *et al*. (2022); Yelimlieş *et al*. (2023)
			signal flexibility	Birds	**+**	greater acoustic signal flexibility	Gentry *et al*. (2017)
					**0**	no difference in acoustic signal flexibility	Potvin & Mulder (2013)
			vocal complexity	Mammals	**+**	more complex	Starik & Göttert (2022)
				Birds	**+**	more complex	Moseley *et al*. (2019)
					**−**	less complex	Smith‐Vidaurre *et al*. (2021)
					**0**	no difference in complexity	Hill *et al*. (2018); Brewer & Fudickar (2022)
			vocal activity	Birds	**+**	vocalise earlier, or more mid‐day	Cartwright *et al*. (2014); Kucera *et al*. (2019); Bermúdez‐Cuamatzin *et al*. (2020)
					**−**	vocalise less mid‐day	Bermúdez‐Cuamatzin *et al*. (2020)
			signal detection	Birds	**+**	greater acoustic signal transmission	Gall *et al*. (2012)
					**−**	reduced acoustic signal transmission	Phillips *et al*. (2020b)
			signalling modality	Mammals	**+**	stronger response to visual signals	Partan *et al*. (2010)
				Birds	**+**	greater use of visual signals	Akçay *et al*. (2020a); Önsal *et al*. (2022)
	**Human activity**	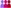	rate of social interactions	Mammals	**+**	increased social interactions	Marty *et al*. (2019); Costa *et al*. (2023a,b)
					**−**	reduced social interactions	Kaburu *et al*. (2019a,b); Thatcher *et al*. (2019); Marty *et al*. (2019); Balasubramaniam *et al*. (2020); Gall *et al*. (2022)
			aggression	Birds	**+**	increased aggression	Colombelli‐Négrel *et al*. (2023); Walters *et al*. (2023)
			acoustic signals	Birds	**+**	increased use of common syllable types	Colombelli‐Négrel *et al*. (2023)
					**0**	no effect on rate or duration	Colombelli‐Négrel *et al*. (2023)
	**Anthropogenic food**	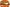	rate of social interactions	Mammals	**+**	increased social interactions	Hodgson *et al*. (2004); Thatcher *et al*. (2019)
					**0**	no effect on affiliative behaviour	Kaburu *et al*. (2019a); Balasubramaniam *et al*. (2020)
			aggression	Mammals	**+**	increased aggression	Hodgson *et al*. (2004); El Alami *et al*. (2012); Theimer *et al*. (2015); Scheun *et al*. (2015)
				Birds	**+**	increased aggression	Foltz *et al*. (2015)
	**Urban structures**	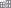	aggression	Mammals	**+**	increased aggression	Parker & Nilon (2012)
				Reptiles	**−**	reduced aggression	Baird *et al*. (2020)
			visual signals	Reptiles	**+**	increased visual displays	Stroud *et al*. (2019)
			acoustic signals	Birds	**+**	higher frequency	Job *et al*. (2016)
					**−**	lower frequency or rate	Dowling *et al*. (2012); Job *et al*. (2016)
					**0**	no effect on frequency, rate, or duration	Dowling *et al*. (2012); Narango & Rodewald (2016); Job *et al*. (2016); Zhan *et al*. (2021)
			vocal performance	Birds	**−**	lower performance	Dowling *et al*. (2012); Job *et al*. (2016)
			signal transmission	Birds	**−**	disrupted acoustic signal transmission	Slabbekoorn *et al*. (2007)
					**0**	no effect on acoustic signal transmission	LaZerte *et al*. (2015)
	**Anthropogenic noise**	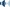	coordination	Mammals	**−**	disrupted behaviour coordination	Sørensen *et al*. (2023)
				Fish	**−**	disrupted shoaling	Herbert‐Read *et al*. (2017)
			rate of social interactions	Birds	**+**	increased sociality	Owens *et al*. (2012)
					**−**	reduced between‐group interactions	Hawkins *et al*. (2020)
				Fish	**+**	increased submissive behaviour	Sebastianutto *et al*. (2011); Bruintjes & Radford (2013); Braga Goncalves *et al*. (2021)
					**−**	reduced affiliative behaviour	Braga Goncalves *et al*. (2021)
			aggression	Birds	**+**	increased aggression	Phillips & Derryberry (2018); LaZerte *et al*. (2019); Wolfenden *et al*. (2019); Grabarczyk & Gill (2019a); Akçay *et al*. (2020b); Önsal *et al*. (2022); Chavez‐Mendoza *et al*. (2023)
					**−**	reduced aggression	Kleist *et al*. (2016); Zwart *et al*. (2016); Passos *et al*. (2020)
				Fish	**+**	increased aggression	Bruintjes & Radford (2013); Mills *et al*. (2020)
			acoustic signals	Mammals	**+**	increased amplitude	Parks *et al*. (2010); Jiang *et al*. (2019); Sørensen *et al*. (2023)
					**+**	increased duration or rate	Sørensen *et al*. (2023)
					**−**	reduced duration	Song *et al*. (2019)
					**0**	no effect on frequency, duration, or rate	Song *et al*. (2019); Jiang *et al*. (2019); Shannon *et al*. (2020)
				Birds	**+**	increased frequency, amplitude, or tonality	Rheindt (2003); Wood & Yezerinac (2009); Mockford & Marshall (2009); Bermúdez‐Cuamatzin *et al*. (2009, 2010); Halfwerk & Slabbekoorn (2009); Verzijden *et al*. (2010); Francis *et al*. (2011a,b); Seger‐Fullam *et al*. (2011); Potvin *et al*. (2011, 2016); Hamao *et al*. (2011); Hanna *et al*. (2011); Proppe *et al*. (2012); Dowling *et al*. (2012); Lowry *et al*. (2012); Luther & Derryberry (2012); McLaughlin & Kunc (2013); Potvin & Mulder (2013); McMullen *et al*. (2014); Luther *et al*. (2016); Narango & Rodewald (2016); Derryberry *et al*. (2016, 2020); LaZerte *et al*. (2016, 2017a); Job *et al*. (2016); Templeton *et al*. (2016); Gentry *et al*. (2017); Davidson *et al*. (2017); Zollinger *et al*. (2017); Warrington *et al*. (2018); Moseley *et al*. (2018); Grabarczyk & Gill (2019a); Sheldon *et al*. (2020); Juárez *et al*. (2021); Winandy *et al*. (2021a); Injaian *et al*. (2021); Zhan *et al*. (2021); Sementili‐Cardoso & Donatelli (2021)
					**+**	increased duration, rate or activity	Halfwerk & Slabbekoorn (2009); Díaz *et al*. (2011); Hamao *et al*. (2011); Francis *et al*. (2011b); Ríos‐Chelén *et al*. (2013); Potvin & MacDougall‐Shackleton (2015); Davidson *et al*. (2017); Grabarczyk & Gill (2019a); Grabarczyk *et al*. (2020b); Courter *et al*. (2020); Vincelette *et al*. (2021); Winandy *et al*. (2021a); Injaian *et al*. (2021); Gallardo Cruz *et al*. (2021); Sánchez *et al*. (2023)
					**−**	decreased frequency or bandwidth	Potvin *et al*. (2011, 2014); Ríos‐Chelén *et al*. (2015); Potvin & MacDougall‐Shackleton (2015); Gentry *et al*. (2017); Wolfenden *et al*. (2019); Liang *et al*. (2020); Courter *et al*. (2020); Winandy *et al*. (2021a); Önsal *et al*. (2022)
					**−**	decreased duration, rate or activity	Potvin *et al*. (2011); Francis *et al*. (2011b); McLaughlin & Kunc (2013); McMullen *et al*. (2014); Ríos‐Chelén *et al*. (2015); Wolfenden *et al*. (2019); Grabarczyk *et al*. (2020b); Gallardo Cruz *et al*. (2021); Önsal *et al*. (2022); Ritz‐Radlinská *et al*. (2023)
					**0**	no effect on frequency or bandwidth	Francis *et al*. (2011a); Hanna *et al*. (2011); Dowling *et al*. (2012); Ríos‐Chelén *et al*. (2013, 2015); Cartwright *et al*. (2014); Job *et al*. (2016); Templeton *et al*. (2016); Gentry *et al*. (2017); Zollinger *et al*. (2017); LaZerte *et al*. (2017a); Gentry & Luther (2019); Akçay *et al*. (2020b); Grabarczyk *et al*. (2020b); Liu *et al*. (2020); Deoniziak & Osiejuk (2021); Sánchez *et al*. (2023)
					**0**	no effect on duration or rate	Mockford & Marshall (2009); Seger‐Fullam *et al*. (2011); Hanna *et al*. (2011); McLaughlin & Kunc (2013); Potvin & Mulder (2013); Cartwright *et al*. (2014); Narango & Rodewald (2016); Job *et al*. (2016); Grabarczyk *et al*. (2020a); Akçay *et al*. (2020b); Winandy *et al*. (2021a); Zhan *et al*. (2021); Deoniziak & Osiejuk (2021); Sánchez *et al*. (2023); Chavez‐Mendoza *et al*. (2023)
				Amphibians	**+**	increased amplitude	Yeo & Sheridan (2019)
					**0**	no effect on frequency or rate	Yeo & Sheridan (2019)
				Crustacea	**+**	increased rate	Jézéquel *et al*. (2021)
			signal flexibility	Birds	**+**	vocal flexibility	Halfwerk & Slabbekoorn (2009); Bermúdez‐Cuamatzin *et al*. (2010); Verzijden *et al*. (2010); Hanna *et al*. (2011); Potvin & Mulder (2013)
					**+/−**	only for noise‐experienced birds	LaZerte *et al*. (2016, 2017a); Gentry & Luther (2019); de Framond & Brumm (2022)
			vocal complexity	Mammals	**−**	reduced complexity	Jiang *et al*. (2019)
				Birds	**+**	switched vocalisation type	Halfwerk & Slabbekoorn (2009); LaZerte *et al*. (2017a)
					**−**	lower complexity, repertoire size, or vocal performance	Verzijden *et al*. (2010); Potvin *et al*. (2011); McLaughlin & Kunc (2013); Redondo *et al*. (2013); Cartwright *et al*. (2014); McMullen *et al*. (2014); Luther *et al*. (2016); Job *et al*. (2016); Davidson *et al*. (2017); Moseley *et al*. (2018); Phillips *et al*. (2020a); Juárez *et al*. (2021); Winandy *et al*. (2021a); Sánchez *et al*. (2023)
					**0**	no effect on song complexity	Cartwright *et al*. (2014); Potvin & MacDougall‐Shackleton (2015); Zhan *et al*. (2021)
			vocal activity	Birds	**+**	vocalise earlier	de Framond & Brumm (2022)
					**+**	vocalise later	Fuller *et al*. (2007); Ritz‐Radlinská *et al*. (2023)
					**0**	no effect on timing of vocal activity	Job *et al*. (2016); Stuart *et al*. (2019); de Framond & Brumm (2022)
			signal detection	Mammals	**−**	reduced detection of acoustic signals	Shier *et al*. (2012); Kern & Radford (2016)
				Birds	**−**	reduced detection or transmission of acoustic signals	Pohl *et al*. (2009 (2012); LaZerte *et al*. (2015); Templeton *et al*. (2016); Grabarczyk & Gill (2020)
				Fish	**−**	reduced detection of acoustic signals	Vasconcelos *et al*. (2007)
			receiver response	Mammals	**−**	false response to noise	Shier *et al*. (2012); Kern & Radford (2016)
				Birds	**+**	stronger response to noise‐adapted songs	Luther & Derryberry (2012); LaZerte *et al*. (2017b)
					**+**	stronger response to songs from similar noise levels	Mockford & Marshall (2009); Sheldon *et al*. (2020)
					**−**	weaker response or discrimination	Luther & Magnotti (2014); Kleist *et al*. (2016); Luther *et al*. (2016); Templeton *et al*. (2016); Damsky & Gall (2017); Montenegro *et al*. (2021)
					**0**	no effect on receiver response	Winandy *et al*. (2021b)
				Amphibians	**−**	false response to noise	Engbrecht *et al*. (2015)
			signalling modality	Mammals	**+**	increased scent‐marking	Sobroza *et al*. (2023)
				Birds	**+**	increased signal redundancy	Akçay & Beecher (2019)
					**0**	no increase in visual displays	Ríos‐Chelén *et al*. (2015); Akçay & Beecher (2019); Önsal *et al*. (2022)
	**ALAN**	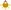	vocal activity	Birds	**+**	vocalise earlier	Kempenaers *et al*. (2010)
					**0**	no effect on timing of vocal activity	Fuller *et al*. (2007); Stuart *et al*. (2019)
	**Chemical pollutants**	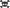	aggression	Birds	**+**	increased aggression in areas with lead pollution	McClelland *et al*. (2019)
**Mating system**	**Urban *vs* non‐urban**	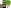	intersex interactions	Reptiles	**−**	fewer intersex interactions	Lacy & Martins (2003)
			visual signals	Reptiles	**−**	reduced colour intensity and slower colour change	Batabyal & Thaker (2017, 2019)
			acoustic signals	Birds	**+**	longer duration	Marini *et al*. (2017)
				Amphibians	**+**	longer duration or higher rate	Halfwerk *et al*. (2019); Grenat *et al*. (2023)
			signal flexibility	Amphibians	**+**	males flexibly adjusted signals	Halfwerk *et al*. (2019); Grenat *et al*. (2023)
			vocal performance	Birds	**+**	higher vocal performance or output	Marini *et al*. (2017); Moseley *et al*. (2019)
					**−**	no longer reflects male quality	Narango & Rodewald (2018)
				Amphibians	**+**	more conspicuous	Halfwerk *et al*. (2019)
			mate preference	Amphibians	**+**	females prefer urban males	Halfwerk *et al*. (2019)
	**Anthropogenic noise**	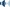	courtship displays	Fish	**−**	reduced courtship displays	de Jong *et al*. (2018a); Vieira *et al*. (2021)
					**0**	no effect on visual displays	de Jong *et al*. (2018a)
			mating behaviour	Birds	**−**	reduced pairing success	Habib *et al*. (2007)
				Fish	**−**	reduced spawning success	de Jong *et al*. (2018a)
				Crustacea	**−**	reduced mating behaviour	Rising *et al*. (2022)
			visual signals	Amphibians	**−**	reduced colouration intensity	Troïanowski *et al*. (2015)
			acoustic signals	Birds	**+**	increased rate or frequency	Slabbekoorn & Peet (2003); LaZerte *et al*. (2017a); Walters *et al*. (2019); Winandy *et al*. (2021a); Barrero *et al*. (2021)
					**−**	shorter duration	Barrero *et al*. (2021)
					**0**	no effect on frequency, rate, or duration	LaZerte *et al*. (2017a); Gentry & Luther (2019); Winandy *et al*. (2021a); Barrero *et al*. (2021)
				Amphibians	**+**	increased frequency or amplitude	Parris *et al*. (2009); Cunnington & Fahrig (2010); Kaiser *et al*. (2011); Kruger & Du Preez (2016); Caorsi *et al*. (2017); Yeo & Sheridan (2019); Leon *et al*. (2019); Grenat *et al*. (2019, 2023); Legett *et al*. (2020); Jiménez Vargas & Vargas Salinas (2021); Higham *et al*. (2021); Zhao *et al*. (2021); Lima *et al*. (2022)
					**+**	increased activity, duration or rate	Sun & Narins (2005); Kruger & Du Preez (2016); Higham *et al*. (2021); Lima *et al*. (2022)
					**−**	decreased frequency or amplitude	Lengagne (2008); Cunnington & Fahrig (2010); Kaiser *et al*. (2011); Lukanov *et al*. (2014); Caorsi *et al*. (2017); Leon *et al*. (2019); Lukanov & Naumov (2019); Legett *et al*. (2020); Zhao *et al*. (2021)
					**−**	decreased activity, duration or rate	Sun & Narins (2005); Lengagne (2008); Cunnington & Fahrig (2010); Leon *et al*. (2019); Lukanov & Naumov (2019); Legett *et al*. (2020); Zhao *et al*. (2021)
					**0**	no effect on frequency, amplitude, rate or duration	Lengagne (2008); Cunnington & Fahrig (2010); Kaiser *et al*. (2011); Troïanowski *et al*. (2015); Nelson *et al*. (2017); Caorsi *et al*. (2017); Yeo & Sheridan (2019); Grenat *et al*. (2019); Jiménez Vargas & Vargas Salinas (2021); Zhao *et al*. (2021); Lima *et al*. (2022)
				Insects	**+**	increased frequency	Lampe *et al*. (2012)
					**−**	decreased frequency and rate	Sathyan & Couldridge (2021)
			signal flexibility	Amphibians	**+**	immediate change in vocal signal	Cunnington & Fahrig (2010)
					**0**	no vocal flexibility	Lengagne (2008)
			vocal performance	Birds	**+**	altered song or syllable type composition	LaZerte *et al*. (2017a); Walters *et al*. (2019)
					**−**	reduced performance	Luther *et al*. (2016); Phillips *et al*. (2020a); Winandy *et al*. (2021a)
			signalling activity	Insects	**+**	signal later	Sathyan & Couldridge (2021)
			signal detection	Birds	**−**	reduced detection or discrimination	Halfwerk *et al*. (2012); Grabarczyk & Gill (2019b); Montenegro *et al*. (2021)
				Amphibians	**−**	reduced female response	Bee & Swanson (2007)
					**−**	reduced transmission	Nelson *et al*. (2017)
				Fish	**−**	reduced detection	Vieira *et al*. (2021)
				Crustacea	**0**	no effect on response time	Rising *et al*. (2022)
				Insects	**−**	reduced female response	Schmidt *et al*. (2014)
			mate preference	Birds	**−**	reduced female response to low‐frequency songs	Halfwerk *et al*. (2011); Huet des Aunay *et al*. (2014)
				Amphibians	**−**	females preferred unattractive calls	Schou *et al*. (2021)
				Fish	**+**	females increased attention to visual signals	de Jong *et al*. (2018b)
			mate attraction	Amphibians	**0**	no effect on mate attraction	Cunnington & Fahrig (2013)
	**Urban structures**	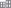	courtship displays	Reptiles	**+**	increased display rate	Baird *et al*. (2020)
	**ALAN**	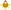	vocal activity	Birds	**+**	vocalise earlier	Kempenaers *et al*. (2010)
			mate preference	Insects	**−**	reduced female discrimination	Botha *et al*. (2017)
			mating behaviour	Birds	**+**	increased extra‐pair paternity	Kempenaers *et al*. (2010)
				Insects	**+**	increased mating success	Botha *et al*. (2017)
**Care system**	**Urban *vs* non‐urban**	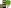	family living	Birds	**+**	family units more common	Luna *et al*. (2021)
			parental care	Birds	**0**	no trade‐off between aggression and parental care	Lane & Sewall (2022)
	**Anthropogenic noise**	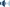	parental defence	Fish	**−**	reduced anti‐predator defence	Bruintjes & Radford (2013)
**Interspecific interactions**	**Urban *vs* non‐urban**	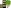	aggression	Birds	**+**	more aggressive	Sedláček *et al*. (2004); Farwell & Marzluff (2013); Fountain & McDonald (2022)
					**−**	less aggressive	Henger & Hauber (2014)
			social information	Birds	**0**	no difference in social information use	Cavalli *et al*. (2018)
	**Anthropogenic noise**	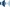	mutualism	Fish	**−**	impaired mutualistic relationship	Nedelec *et al*. (2017); McCloskey *et al*. (2023)
	**Anthropogenic food**	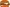	aggression	Fish	**+**	increased aggression	Mills *et al*. (2020)
			social information	Mammals	**−**	reduced response to alarm calls	Morris‐Drake *et al*. (2017)
				Birds	**−**	reduced response to alarm calls	Grade & Sieving (2016); Damsky & Gall (2017)
			aggression	Mammals	**+**	increased aggression	Theimer *et al*. (2015)

## IV. SOCIAL SYSTEMS IN URBAN ENVIRONMENTS

**Fig. 2:**
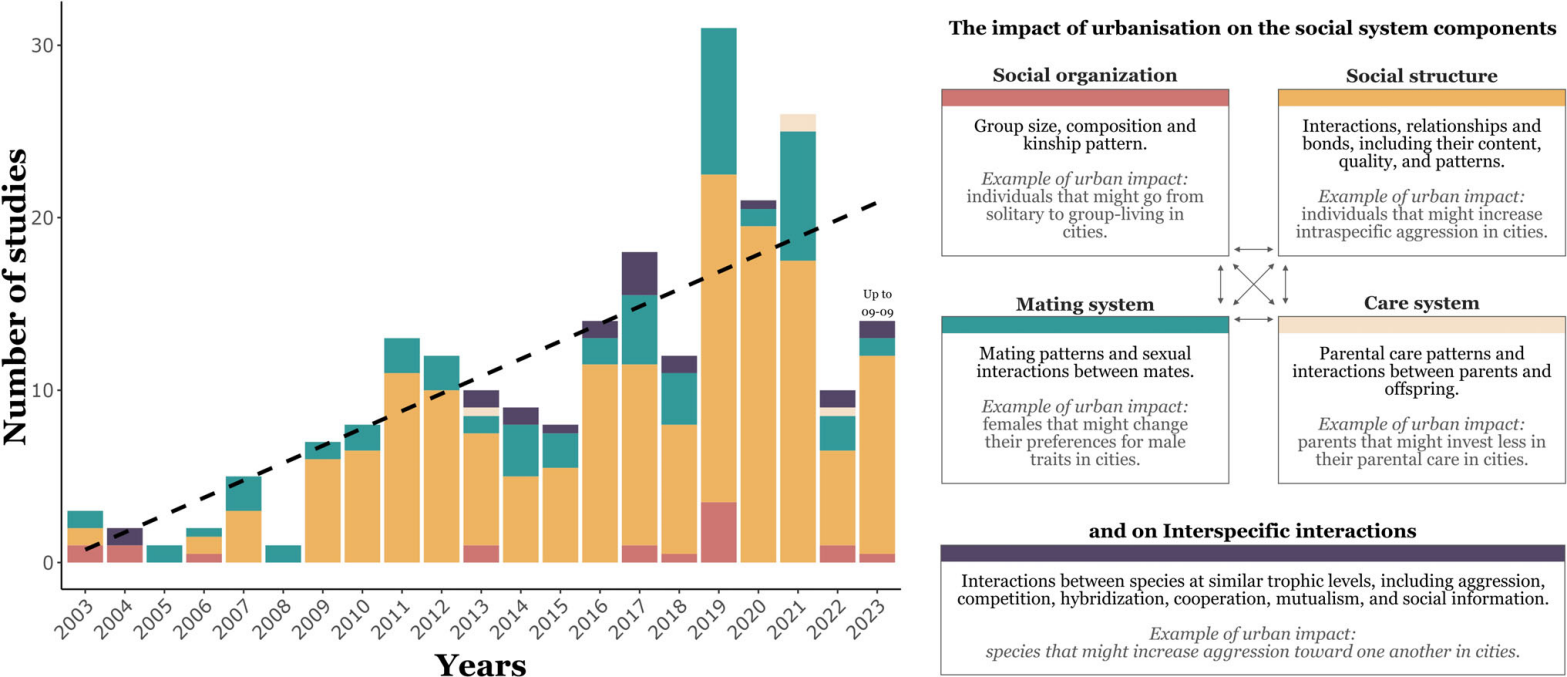
Number of studies investigating the impact of urbanisation on social behaviour (2003–2023), retrieved from our systematic search (see Table 1). Studies are grouped by colours according to the studied social system component, with the respective definition provided on the right (based on Kappeler, 2019). For studies addressing two topics simultaneously (*N* = 25), a value of 0.5 was assigned to each topic for visualisation purposes only.

While it is important to understand how individual urban stressors drive changes in social behaviour, animals living in urban environments must contend with multiple stressors simultaneously, which can lead to different behavioural outcomes. Following Kappeler's (2019) framework, we discuss how urbanisation can impact each of the four components of an animal's social system: (*i*) social organisation, (*ii*) social structure, (*iii*) mating system, and (*iv*) care system (Fig. 2; see Table 1, for summary of included studies and outcomes). We focus primarily on studies comparing urban and non‐urban populations, which can provide an empirical understanding of how social behaviour changes within urban environments. While social systems are inherently complex and these components are often interconnected, this structure serves as a useful guide for discussing the effects of urbanisation on social behaviour. Our literature search identified 75 studies that compared social behaviour between urban and non‐urban populations. We again focus on examples from these studies but include additional work where necessary to provide a more complete overview of how social systems respond to urbanisation. At the end of each section, we summarise the effects of the urban stressors discussed above, offer predictions for how each social component might change as urban areas expand, and suggest directions for future research.

### (1) Social organisation

#### (a) *Urban* versus *non‐urban*

Social organisation describes group‐level characteristics, including size, composition, and kinship patterns (Kappeler, 2019). Social groups range on different levels of stability and complexity, from highly stable eusocial insect colonies to more loose aggregations or fission–fusion societies. Group size and membership can be highly dynamic and change according to local environmental factors (Couzin & Laidre, 2009; Newsome *et al*., 2013). While group formation is associated with benefits such as enhanced resource acquisition and predator defence (Krause & Ruxton, 2002), conflict within groups can arise as group members compete for access to resources and mating opportunities. If resources in urban habitats are more abundant and predictable, individuals may be less reliant on social information to locate resources (Morand‐Ferron *et al*., 2019), potentially leading to smaller group sizes (Kucera *et al*., 2019) or less‐stable group membership (Struller *et al*., 2022). For example, northern cardinals (*Cardinalis cardinalis*) formed smaller non‐breeding groups in urban areas, which was attributed to a high prevalence of bird seed feeders (Kucera *et al*., 2019). Alternatively, the predictive nature of urban resources can free up temporal and energetic constraints on social behaviour, and clumped resource distributions can promote the formation of larger social groups or aggregations. Bushbabies (*Galago moholi*) are typically solitary; however, individuals in urban populations were observed to form permanent social groups of up to 10 individuals and invest more time in social interactions (Scheun *et al*., 2019). Similarly, urban spotted hyenas (*Crocuta crocuta*) were found to exhibit different grouping behaviour than is typical of non‐urban clans, gathering in larger numbers to forage in an urban waste dump, without discernible group borders (Struller *et al*., 2022).

Social groups are composed of individuals who can differ in their movement patterns and motivations. Environmental variability in urban spaces can exacerbate these differences, leading to reduced cohesion within groups. For example, individuals can differ in their risk tolerance and willingness to access urban areas (Morrow *et al*., 2019; Satsias *et al*., 2022). Habitat fragmentation and structural barriers in urban habitats can further reduce group cohesion by visually isolating individuals and impeding collective movement (Banks *et al*., 2007). Individuals within a group of chacma baboons (*Papio ursinus*) became more dispersed, formed smaller sub‐groups, and exhibited reduced cohesion when foraging in urban areas (Bracken *et al*., 2022). Disruption to group size and movement can have cascading effects on behaviour as individuals will need to invest more time in re‐establishing relationships with group members, and even temporary changes in group size or composition can impair group functioning (Maldonado‐Chaparro *et al*., 2018), and have fitness costs (Ebensperger *et al*., 2017).

Selection pressures can act differently according to phenotype (Höjesjö *et al*., 2004), and animals can show intraspecific variation in their response to urbanisation (Harding *et al*., 2019), potentially altering the composition of urban social groups. For example, increased farming activity disproportionately killed female whinchats (*Saxicola rubetra*), which altered the adult sex ratio, and ultimately led to population declines (Grüebler *et al*., 2008). Groups of urban white‐winged choughs (*Corcorax melanorhamphos*) also have fewer adult females compared to non‐urban groups, although the cause was unclear (Beck & Heinsohn, 2006). In addition to sex‐specific effects, selection pressures can differently impact individuals according to age, body condition, social status, or personality, which can ultimately shift the phenotypic composition of a social group or population (Sapolsky & Share, 2004), with consequences for group‐level outcomes (Farine, Montiglio & Spiegel, 2015).

#### (b) Impact of urban stressors

Environmental stressors inherent to urbanisation can greatly influence social organisation by impacting the individuals that form social groups, which can in turn affect emergent group traits. Exposure to lethal chemical pollutants can influence the number and diversity of animals available to interact with (Michelangeli *et al*., 2022), while higher temperatures in cities can bias sex ratios in species with temperature‐dependent sex determination (Mitchell & Janzen, 2010; Blumstein *et al*., 2023). The distribution of anthropogenic food resources in the environment can influence grouping patterns (Jiménez *et al*., 2013; Scheun *et al*., 2019; Struller *et al*., 2022). Human activity can disrupt social organisation by limiting the time spent on social interactions and maintaining relationships, leading to reduced group cohesion (Kaburu *et al*., 2019a,b; Balasubramaniam *et al*., 2020). Anthropogenic noise can impede group movements and collective behaviour by disrupting communication (Herbert‐Read *et al*., 2017), while urban structures can create barriers that further limit cohesive group movements, as well as limit the number and composition of individuals available to interact with (He, Maldonado‐Chaparro & Farine, 2019). Environmental stressors that disproportionately target certain individuals can shift group composition or result in the loss of key individuals in a social group, which can hinder group movement and decision making (Wheatcroft & Price, 2018) or reduce social stability (Flack *et al*., 2006; Williams & Lusseau, 2006).

#### (c) Predictions and outlook

Urbanisation is likely to promote more dynamic and loosely structured social groups, with the direction and magnitude of change influenced by a species' intrinsic social tendencies. In obligately group‐living species, urban stressors that interfere with cohesion, collective movement, or communication may weaken group stability and function. This breakdown in cohesion is likely to lead to smaller, less stable groups, particularly in species that rely on strong social bonds for group functioning. By contrast, for facultatively social or more solitary species, spatial constraints and altered resource distributions are likely to increase the frequency and size of social aggregations. These social groupings may be less dependent on relatedness or social bonds. Restricted dispersal in urban habitats may increase relatedness within urban groups, although this has not been well addressed. At the same time, shifts in sex ratios and selection pressures will likely alter the demographic and phenotypic composition of social groups, with implications for group functioning. Social organisation in urban environments may increasingly reflect a trade‐off between ecological conditions that promote aggregations and urban stressors that disrupt and undermine group cohesion.

Our literature search retrieved only 14 studies (6%) that assessed the impact of urbanisation on social organisation and were biased towards mammals and group‐living species. However, species that are typically ‘less social’ may still exhibit changes in social organisation, and researchers should strive to include a broader range of taxa. Further research is needed to understand how urbanisation alters group composition, particularly with respect to relatedness, sex ratios, and trait‐based filtering. Studies that track how group membership changes across urban gradients, or that compare demographic and phenotypic diversity between urban and non‐urban groups, could offer valuable insights.

### (2) Social structure

#### (a) *Urban* versus *non‐urban*

Social structure encompasses social interactions between conspecifics (including communication) and arises from repeated interactions between individuals (Kappeler, 2019). While Kappeler (2019) indicates that social structure refers to interactions between individuals within the same social unit, we include interactions that may not necessarily occur within a social unit, such as territorial aggression. Furthermore, while communication signals can be used in different and overlapping contexts that can be difficult to disentangle, we attempt to focus on intrasexual communication in this section and discuss sexual signalling in the ‘mating system’ section (Section IV.3). Below, we discuss how patterns of aggression, tolerance, within‐group behaviour, and communication can differ between urban and non‐urban habitats.

Urban environments differ in their habitat structure and resource abundance relative to non‐urban habitats, and often support higher population densities, which can significantly influence patterns of aggression and tolerance in urban animals. Studies that compared urban and non‐urban populations of water dragons (*Intellagama lesueurii*) (Baxter‐Gilbert & Whiting, 2019) and towhees (*Pipilo aberti*) (Fokidis, Orchinik & Deviche, 2011) found that urban individuals were more aggressive, which was associated with higher population densities. However, male song sparrows (*Melospiza melodia*) were also found to be more aggressive in urban habitats despite lower population densities (Davies & Sewall, 2016), suggesting other factors may be promoting heightened aggression in some urban populations. One possibility is that, if urban habitats are perceived as higher quality, heightened aggression is selected for, as only the most aggressive individuals are able to secure and defend territories. In support of this, urban curve‐billed thrashers (*Toxostoma curvirostre*) were found to display more territorial aggression when defending territories containing desert vegetation, which is a preferred nesting site (Fokidis *et al*., 2011).

Limited habitat space and high population densities are expected to increase the frequency of social encounters and the costs of social interactions. Becoming more socially tolerant towards conspecifics may therefore be a more adaptive strategy for some urban species, given the costs of engaging in aggressive behaviour. A study using camera traps found that urban populations of striped field mice (*Apodemus agrarius*) were more tolerant and less likely to avoid close contact with conspecifics than non‐urban populations (Łopucki, Klich & Kiersztyn, 2021). Similarly, common wall lizards (*Podarcis muralis*), a typically territorial species, exhibit greater social tolerance and formed more social connections in urban habitats (Maune *et al*., 2025). Such changes in tolerance may reflect behavioural adjustments that reduce conflict where encounters are more frequent. Furthermore, if resources are more abundant in urban habitats, individuals may be less motivated to compete aggressively with conspecifics, as observed in urban male house finches (*Haemorhous mexicanus*) (Hasegawa *et al*., 2014, 2018).

Structural barriers in urban environments limit movement and dispersal for many species, forcing animals to share habitat space at higher densities (Mitrovich *et al*., 2009). This increased proximity may promote the formation of dominance hierarchies, which arise through repeated competitive interactions between individuals. Being aware of the social environment (i.e. social competence) (Taborsky & Oliveira, 2012) and adjusting behaviour accordingly may reduce conflict in urban habitats where individuals are forced into more stable groupings due to limited dispersal (Fisher *et al*., 2021). Indeed, high population density has been found to induce the formation of dominance hierarchies in typically territorial species (Alberts, 1994). However, environmental stressors can also disrupt these hierarchies (Fisher *et al*., 2021), and more research is needed to understand how urbanisation influences their formation and maintenance.

For group‐living species, the strength of social relationships between group members can be dynamic or cyclical, and vary according to environmental conditions that are affected by urbanisation (Henzi *et al*., 2009). For example, consistent access to food in urban environments can free up time for investing in social relationships (Jaman & Huffman, 2013) or reduce competition between group members, which can in turn strengthen cohesion within groups (Scheun *et al*., 2015). Alternatively, patchy resources in urban areas may reduce tolerance (Flint *et al*., 2016) and weaken social bonds between group members (Belton *et al*., 2018). Indeed, environmental variability in urban habitats can exacerbate intragroup conflict and reduce social stability, which in turn may impair collective decision‐making and movement (Bracken *et al*., 2022). An experimental study that temporarily split social groups of zebra finches (*Taeniopygia guttata*) demonstrated that even temporary disruptions to social stability can weaken social bonds and impair a group's ability to exploit food patches (Maldonado‐Chaparro *et al*., 2018). Thus, group‐living animals may face fitness costs in urban environments that promote social instability.

Communication plays an important role in facilitating other social and reproductive behaviours. Consequently, communication signals are likely facing strong selective pressures in urban habitats. Indeed, communication in cities has been relatively well studied, and there is substantial evidence that patterns and signals differ between urban and non‐urban populations (Mockford & Marshall, 2009; Nemeth & Brumm, 2009; Hu & Cardoso, 2009; Potvin *et al*., 2011; Nemeth *et al*., 2013; Narango & Rodewald, 2016; LaZerte *et al*., 2017a; Hill *et al*., 2018; Bermúdez‐Cuamatzin *et al*., 2020; Brewer & Fudickar, 2022). In Section III, we discussed how environmental stressors, such as sensory and chemical pollutants, can disrupt communication. However, communication occurs within a social context, and biotic conditions in urban environments can also exert significant selection pressures on communication patterns (Hamao, Watanabe & Mori, 2011; Stuart *et al*., 2019; Shannon *et al*., 2020; Grabarczyk, Vonhof & Gill, 2020b; Deoniziak & Osiejuk, 2021). For example, in northern cardinals (*Cardinalis cardinalis*), higher densities in urban habitats were associated with males singing longer and faster songs, which are song characteristics related to conspecific interactions (Narango & Rodewald, 2016). This may be due to increased territorial aggression and more frequent encounters with intruders in urban settings. Conversely, high population densities in urban areas could reduce the intensity of signalling in other species (Batabyal & Thaker, 2017).

Signals often convey information about the signaller's motivation or quality, and those adjusted for urban environments may no longer provide accurate or reliable information (Narango & Rodewald, 2018) and elicit different responses from receivers (Mockford & Marshall, 2009). For example, white‐crowned sparrows (*Zonotrichia leucophrys nuttalli*) and northern cardinals (*Cardinalis cardinalis*) do not respond as strongly to songs adjusted for anthropogenic noise (Luther & Magnotti, 2014; Luther *et al*., 2016), while mountain chickadees (*Poecile gambeli*) respond more aggressively (LaZerte, Slabbekoorn & Otter, 2019). The ability of both senders and receivers to adjust their behaviour to optimise communication may be a determining factor in which species are able to persist in cities.

#### (b) Impact of urban stressors

Impervious urban structures and anthropogenic noise can disrupt communication by impairing the transmission and detection of signals (Vasconcelos *et al*., 2007; Pohl *et al*., 2009; Templeton *et al*., 2016; Nelson *et al*., 2017; LaZerte *et al*., 2019; Grabarczyk & Gill, 2020), which can in turn influence aggression (Phillips & Derryberry, 2018; Grabarczyk & Gill, 2019a; Akçay, Beck & Sewall, 2020a; Akçay *et al*., 2020b; Önsal *et al*., 2022) and group behaviour (Hawkins *et al*., 2020). Human activity can impose temporal constraints on social interactions (Kaburu *et al*., 2019a; Balasubramaniam *et al*., 2020; Gall *et al*., 2022) or promote social behaviours that strengthen social bonds and reduce physiological stress (Costa *et al*., 2023a,b). Access to anthropogenic food can allow animals to invest more in social relationships by freeing up time otherwise spent foraging (Jaman & Huffman, 2013) or lead to increased aggression and competition (Theimer *et al*., 2015; Flint *et al*., 2016). Exposure to chemical pollutants can impact aggressive behaviour (McClelland *et al*., 2019) and disrupt dominance hierarchies (Sopinka, Marentette & Balshine, 2010).

#### (c) Predictions and outlook

The impact of urbanisation on social structure is likely to depend on both ecological context and species‐specific traits such as social tendencies and movement ecology. In solitary or territorial species, high population densities and spatial constraints will likely increase opportunities for social encounters, promoting greater tolerance or facilitating the emergence of dominance hierarchies when dispersal is limited. By contrast, species with greater dispersal ability or species that depend on territory defence for reproductive success may become more aggressive, especially if urban habitats are viewed as higher quality. While some aspects of the urban environment can strengthen social bonds, urbanisation is ultimately likely to destabilise social groups by increasing conflict between group members. Species with greater social flexibility (Taborsky & Oliveira, 2012) should be more resilient to urban‐driven changes in social structure than those with more specialised social systems.

Social structure is by far the best studied component of an animal's social system and comprises 75% (*N* = 170) of the studies from our literature search. While social structure encompasses a range of important behaviours, most of these studies were focused on communication (71%, *N* = 121). There is substantial evidence that urban environments can drive rapid shifts in communication strategies. However, whether these adjustments maintain signal function for both senders and receivers remains uncertain. Further research is needed on other aspects of social structure, such as the rate of social encounters, formation of dominance hierarchies, and within‐group social dynamics, which can influence individual fitness, group stability, and population‐level outcomes.

### (3) Mating system

#### (a) *Urban* versus *non‐urban*

A species' mating system describes who mates with whom and includes mating patterns and reproductive tactics (Kappeler, 2019). We include courtship behaviour and sexual signalling in this section. Like other social behaviours, mating behaviours are influenced by local environmental conditions and likely face strong selective pressures in urban environments, given their direct impact on reproductive output (Sepp, McGraw & Giraudeau, 2020; Cronin *et al*., 2022b).

Effective sexual signalling depends on clear signal transmission, which may be constrained in urban environments, and animals should therefore adjust their signals to optimise transmission and detection [for reviews on how urbanisation affects sexual signalling, see Heinen‐Kay *et al*. (2021) and Cronin *et al*. (2022b)]. However, sexual signals adjusted for urban environments may no longer convey accurate information about the quality of the sender (Narango & Rodewald, 2018) and can conflict with established mate choice preferences. For example, in many bird species, females prefer males with low‐frequency songs, which are more likely to be masked by anthropogenic noise (Halfwerk *et al*., 2011; Huet des Aunay *et al*., 2014). If sexually attractive signals are less detectable in urban environments, sexual selection may shift to favour characteristics that enhance signal transmission (Halfwerk *et al*., 2011; Huet des Aunay *et al*., 2014; Schou, Levengood & Potvin, 2021). Biotic conditions, such as predation pressure and population density, can also exert selection on sexual signals in urban environments. For example, high population densities can weaken sexual selection pressures by increasing the number of available mates (Kokko & Rankin, 2006). Urban male Indian rock agamas (*Psammophilus dorsalis*) displayed lower courtship intensity and less‐vibrant colours compared to non‐urban males, which was attributed to lower motivation due to the greater availability of females (Batabyal & Thaker, 2017, 2019). Conversely, urban male túngara frogs (*Physalaemus pustulosus*) produced more complex and conspicuous mating calls in response to increased competition for mates and reduced predation pressure (Halfwerk *et al*., 2019). Urban males were found to attract significantly more females than non‐urban males, even under the same noise and light conditions, suggesting that urban signals were indeed adaptive (Halfwerk *et al*., 2019). Further studies comparing urban and non‐urban sexual signals in differing conditions can provide insight into whether urban phenotypes are adaptive and identify the strength and direction of sexual selection (Cronin *et al*., 2022b; Kuriwada, 2023).

The habitat configuration and distribution of resources in urban areas can shift the spatio‐temporal distribution of receptive mates (Emlen & Oring, 1977) and may drive the emergence of alternate mating strategies (Schradin *et al*., 2012; Sih *et al*., 2017). Remarkably, bobucks (*Trichosurus cunninghami*) living in a forested area where resources were dispersed are monogamous, while bobucks living along a road with more clumped resources exhibit polygyny, with male home ranges overlapping with multiple females (Martin & Martin, 2007). Similarly, the structural complexity of urban habitats can influence the likelihood of encountering potential mates by shifting movement patterns. For example, in a human‐modified habitat that was structurally simple, territorial male collared lizards (*Crotaphytus collaris*) moved more and experienced more encounters with females, resulting in greater reproductive success, compared to individuals living in a more structurally complex habitat (Braun, Baird & York, 2018). However, urbanisation may also impose barriers that limit movement and constrain the ability of animals to locate potential mates and can potentially lead to greater reproductive variance (Banks *et al*., 2007) and reduced genetic diversity (Riley *et al*., 2014; López‐Uribe *et al*., 2015). In Cuban rock iguanas (*Cyclura nubila*), males typically interact with one female, however, in an urban population, 40% of males did not interact with any females, while some males interacted with more than four (Lacy & Martins, 2003). This shift in reproductive variance can ultimately increase the risk of inbreeding in urban populations. Higher population densities in urban habitats may also act as a switch point between different reproductive tactics (Kokko & Rankin, 2006). For example, under high population densities, territory defence can become too costly, and animals may switch to alternative reproductive strategies such as dominance polygyny, scramble competition, or lekking (Langbein & Thirgood, 1989; Kokko & Rankin, 2006).

Animals can also adjust their reproductive timing and investment depending on environmental conditions and resource availability (Stephens *et al*., 2024). In some cases, urban habitats may act as a buffer against fluctuating conditions if, for example, access to anthropogenic resources provides a consistent source of energy. This may allow animals to lengthen their breeding season, as observed in white‐winged choughs (*Corcorax melanorhamphos*), which initiated breeding earlier in urban areas (Beck & Heinsohn, 2006). Similarly, urban side‐blotched lizards (*Uta stansburiana*) showed less variation in reproductive investment between dry and rainy years compared to non‐urban populations, which was attributed to irrigation from urban landscaping mitigating the effects of low rainfall (Smith *et al*., 2024). However, this does not necessarily translate into enhanced reproductive success as urban choughs were also found to suffer more nest failures and had lower fledgling survival (Beck & Heinsohn, 2006). Furthermore, if reproductive timing becomes less synchronous, this can lead to greater reproductive variance (Say, Pontier & Natoli, 2001).

#### (b) Impact of urban stressors

Environmental conditions inherent to urban environments can disrupt reproductive behaviour by altering the traits used to attract, locate, or assess the quality of potential mates. Sensory and chemical pollutants can impair the transmission and detection of acoustic (Bee & Swanson, 2007; Huet des Aunay *et al*., 2014; Schmidt, Morrison & Kunc, 2014; de Jong *et al*., 2018a,b), visual (de Jong *et al*., 2018a), and olfactory (Secondi *et al*., 2009; Rising *et al*., 2022) sexual signals. This can potentially weaken mate discrimination (Botha *et al*., 2017), shift sexual selection (Halfwerk *et al*., 2011; Huet des Aunay *et al*., 2014; Schou *et al*., 2021), and impair reproductive success (Habib, Bayne & Boutin, 2007). Furthermore, these sensory and chemical pollutants can also affect individual health, which can indirectly impact sexual signals (Troïanowski *et al*., 2015; Grunst *et al*., 2020). Exposure to anthropogenic noise and ALAN can also disrupt the timing and synchrony of reproductive behaviours (Baker & Richardson, 2006; Kurvers & Hölker, 2015; Cronin, Smit & Halfwerk, 2022a), while endocrine‐disrupting chemicals can affect mating systems and influence sexual selection (Söffker & Tyler, 2012; Blocker & Ophir, 2013).

#### (c) Predictions and outlook

Urban environments can impose strong and sometimes conflicting selection pressures on reproductive behaviours. Species with flexible reproductive timing and opportunistic mating strategies will likely be more successful in cities, particularly where access to anthropogenic resources buffers seasonal variation. For some species, higher population densities and clumped resource distributions may limit the feasibility of maintaining exclusive territories and promote alternative reproductive tactics. Urban‐induced changes in sex ratios, dispersal, or group composition may further limit mate availability and reduce genetic diversity (López‐Uribe *et al*., 2015; Miles *et al*., 2019). Multi‐modal sexual signalling should be favoured to increase signal detection and redundancy in urban soundscapes (Bro‐Jørgensen, 2010). However, changes in signal structure may conflict with established mate choice preferences, with potential consequences for mate assessment and sexual selection. Species capable of adjusting both signal production and reception may be better able to maintain reproductive success in urban conditions.

23% (*N* = 52) of the studies from our literature search assessed the impact of urban habitats or urban stressors on mating systems. However, like social structure, this was biased towards studies on sexual communication (79%; *N* = 41). While sexual signalling represents an important aspect of reproductive success, additional studies are needed to understand how broader mating strategies and reproductive tactics change in response to urban selection pressures. Future research should explore whether urbanisation drives divergence in mating tactics and courtship interactions, and whether these behavioural changes contribute to reproductive skew, reduced synchrony, or the potential for local adaptation in urban populations.

### (4) Care system

#### (a) *Urban* versus *non‐urban*

A species' care system describes the form of parental care provided to offspring and who provides it (Kappeler, 2019). Investment in parental care varies widely across taxa, and although it can be an important component of reproductive success for many species, there is limited research on how care systems differ between urban and non‐urban habitats. Parental care reflects a trade‐off between investment in current and future reproductive success (Clutton‐Brock & Scott, 1991), and parents should adjust their care behaviour based on environmental conditions such as habitat quality, predation risk, and resource availability (Alonso‐Alvarez & Velando, 2012; Mahr, Riegler & Hoi, 2015). Several studies on birds found that parents invest more in parental care in urban habitats (Estes & Mannan, 2003; Isaksson & Andersson, 2007; Sinkovics *et al*., 2021; Hope, Hopkins & Angelier, 2022). For example, great tits (*Parus major*) (Isaksson & Andersson, 2007; Sinkovics *et al*., 2021) and Cooper's hawks (*Accipiter cooperii*) (Estes & Mannan, 2003) exhibit higher rates of offspring provisioning in urban populations which was attributed to greater prey availability. Protecting offspring against predators is another common form of parental care that can be extremely costly. A comparative study of nest defence among four bird species along a gradient of human impact found that northern cardinals (*Cardinalis cardinalis*) decreased nest defence in more developed areas, while brown thrashers (*Toxostoma rufum*) showed the opposite. American robins (*Turdus migratorius*) and gray catbirds (*Dumetella carolinensis*), two common urban species, showed no relationship between nest defence and urban development (Merrill *et al*., 2016). This variation in parental investment was hypothesised to reflect differences in perceived habitat quality, as parents may be more motivated to invest in their offspring in higher‐quality habitats. Furthermore, urban animals that exhibit other behavioural modifications, such as heightened aggression, may face a trade‐off with parental care behaviours, although this was not observed in female song sparrows (*Melospiza melodia*) (Lane & Sewall, 2022).

In species with bi‐parental care, sexual conflict can arise as parental care is costly, and either parent may be tempted to exploit the other by performing less care (Trivers, 1972). Coordinating care by alternating or synchronising behaviours can reduce this conflict (Bebbington & Hatchwell, 2016) but may be constrained in urban environments. For example, many bird species rely on acoustic communication to coordinate behaviour (Mariette, 2019), which may be masked by anthropogenic noise. Additionally, differences in resource availability or distribution in urban habitats can disrupt synchronised care behaviour by forcing parents to forage separately, as observed in urban house wrens (*Troglodytes aedon*) (Baldan & Ouyang, 2020).

In some species, sexually mature individuals can delay dispersal to remain as subordinates in their natal group, sometimes helping with the care of future offspring (Cant, 2012). The decision to delay dispersal is hypothesised to arise in part due to constraints in finding or obtaining suitable breeding sites (‘ecological constraints hypothesis’; Emlen, 1982), when there is high environmental variability (Rubenstein & Lovette, 2007; Griesser *et al*., 2017), or when delayed dispersers stand to inherit high‐quality breeding sites [‘benefits of philopatry hypothesis’ (Koenig *et al*., 1992; Pasinelli & Walters, 2002)]. A study on burrowing owls (*Athene cunicularia*) found that males sometimes delayed dispersal and remained in their natal nest, which, although rare, was more common in urban habitats (Luna *et al*., 2021). While delayed dispersers did not directly contribute to parental care, their presence in the nest was positively associated with offspring body condition, and delayed dispersers were more likely to inherit high‐quality breeding territories close to their natal nest (Luna *et al*., 2021). If urban habitats are perceived as higher quality or impose high costs to dispersal, delayed dispersal may become more prevalent in urban populations, and, over time, promote different reproductive strategies, such as alloparental care or cooperative breeding. On the other hand, alloparental care may be reduced in urban areas if groups experience greater social instability (Ebensperger *et al*., 2017).

#### (b) Impact of urban stressors

Urban stressors can pose temporal and energetic constraints that may limit parental care. For example, by masking acoustic communication, anthropogenic noise can limit the ability of parents to communicate with their offspring and coordinate care behaviours with each other, which may ultimately increase sexual conflict within pairs (Mariette, 2019). ALAN can alter perceived photoperiods that mediate parental care behaviours (Titulaer *et al*., 2012). Exposure to anthropogenic noise can act as a distraction and reduce predator defence behaviour (Bruintjes & Radford, 2013).

#### (c) Predictions and outlook

Overall, urban environments will likely shift the balance of parental investment by altering the relative costs and benefits of caring for current *versus* future offspring. For species with bi‐parental or cooperative care, disruptions to communication and coordination are likely to increase sexual and social conflict, weakening care partnerships (Mariette, 2019; Moss & Moore, 2021). These effects may be particularly pronounced in species with sexually dimorphic roles, as males and females can be differentially affected by urban stressors. If population density is higher in urban areas, males may be more prone to desert offspring in favour of future mating opportunities (Kokko & Rankin, 2006), especially when the breeding season is extended (Griggio, 2015; Zheng, Komdeur & Weissing, 2023). Conversely, constraints on dispersal in urban habitats may prolong parent–offspring associations and promote the emergence of cooperative care behaviours. Future research could test whether urban environments favour certain care strategies, and under what ecological or social conditions these shifts occur.

Our literature search retrieved only three studies (1%) that assessed the impact of urbanisation on the care system, although we identified six additional studies that addressed offspring provisioning in urban birds, emphasising the importance of using appropriate key words to connect studies between different social systems. Despite this, there is still relatively little information on how care systems change in response to urbanisation. This represents an important area of research that warrants further attention, given the importance of parental care in determining reproductive success, which is essential for species persistence in urban areas. More broadly, understanding how care strategies shift in response to rapid environmental change could offer new insights into the evolution of different care systems.

## V. IMPACT OF URBANISATION ON INTERSPECIFIC INTERACTIONS

In urban spaces, animals not only interact with conspecifics but also are part of interspecific interaction networks. Importantly, other species can influence an animal's ability to persist in an urban habitat (Berg *et al*., 2010), but this has rarely been addressed. Urbanisation can drastically alter multi‐species interaction networks, as many species are unable to survive in cities, while invasive species and ‘urban exploiters’ can become more frequent (McKinney, 2006; Carlon & Dominoni, 2024). Here we discuss how different types of interspecific interactions, including aggression, competition, hybridisation, cooperation, mutualism, and social information use, may be affected by urbanisation.

Urbanisation can create new biotic compositions, as species that do not typically overlap in space use or resources may end up occupying the same habitat in urban areas or exploiting the same anthropogenic food sources. This can ultimately lead to different patterns of aggressive and competitive interactions between species (Farwell & Marzluff, 2013; Theimer *et al*., 2015; Fountain & McDonald, 2022). For example, anthropogenic food may attract multiple species (Theimer *et al*., 2015) and lead to increased interspecific aggression (Fountain & McDonald, 2022). Noisy miners (*Manorina melanocephala*) are more aggressive towards heterospecifics in areas where they have access to anthropogenic food (Fountain & McDonald, 2022). It is worth noting that conditions that promote heightened intraspecific aggression, such as increased stress hormones, may also extend to aggression towards heterospecifics (Mills *et al*., 2020). Moreover, urbanisation can bring together closely related species that are potentially able to hybridise. Black redstarts (*Phoenicurus ochruros*) interact aggressively with common redstarts (*Phoenicurus phoenicurus*) in urban areas despite not overlapping in habitat use or territories, and this increase in aggression was hypothesised to serve as a pre‐copulation barrier (Sedláček, Fuchs & Exnerová, 2004). However, urbanisation can also promote hybridisation by breaking down other barriers to copulation, such as species recognition (Fisher, Wong & Rosenthal, 2006; Grabenstein & Taylor, 2018), which may ultimately reduce reproductive success and fitness if hybrid offspring are sterile or ill‐adapted (Todesco *et al*., 2016).

Cooperative and mutualistic relationships may be particularly sensitive to urbanisation, as urban stressors that act on one species will also indirectly affect its partner and can lead to the breakdown of relationships that may be necessary for fitness or survival (Toby Kiers *et al*., 2010; Irwin *et al*., 2020). Animals should abandon mutualistic relationships when the costs outweigh the benefits, and changing environmental conditions have the potential to shift from mutualistic relationships to exploitative or antagonistic ones. A classic example of a mutualistic relationship is cleaner fish that remove parasites and dead skin from client fish, however, anthropogenic noise, from motorboats or scuba divers, may distract one or both parties and disrupt this behaviour (Nedelec *et al*., 2017; McCloskey *et al*., 2023). Bluestreak cleaner wrasses (*Labroides dimidiatus*) were less cooperative towards their clients and increased cheating behaviour when exposed to anthropogenic noise. However, the client fish did not change their behaviour in response, suggesting they were distracted (Nedelec *et al*., 2017). Changes in community composition, which are usual in urban environments, can lead to the loss of mutualistic partners and may promote partner‐switching (Toby Kiers *et al*., 2010).

Animals can use social information from heterospecifics that overlap in their use of resources, habitat, or predators to help navigate their environment (Avarguès‐Weber, Dawson & Chittka, 2013; Damas‐Moreira *et al*., 2018). However, urban stressors may mask social cues and make them undetectable in urban environments. For example, many species eavesdrop on the alarm calls of heterospecifics to alert them of potential risks, which may be masked by anthropogenic noise. Northern cardinals (*Cardinalis cardinalis*) (Grade & Sieving, 2016), black‐capped chickadees (*Poecile atricapillus*) and tufted titmice (*Baeolophus bicolor*) (Herbert‐Read *et al*., 2017) failed to respond to tufted titmice (*Baeolophus bicolor*) alarm calls in the presence of anthropogenic noise. Similarly, dwarf mongooses (*Helogale parvula*) were less likely to flee in response to squirrel alarm calls in the presence of traffic noise (Morris‐Drake *et al*., 2017).

Our literature search retrieved only 12 studies (5%) that addressed the impact of urbanisation on interspecific social interactions (we excluded studies on space use and predation). Interspecific relationships can have important consequences for fitness and survival, and the presence or absence of certain heterospecifics may preclude animals from inhabiting urban areas, while the nature of interspecific interactions can influence responses to multiple urban stressors (Thompson, MacLennan & Vinebrooke, 2018). Understanding how these relationships are changing in urban habitats is an important element in addressing behavioural responses to urbanisation.

## VI. FUTURE DIRECTIONS

### (1) Synthesis of observed and anticipated social responses

**Fig. 3:**
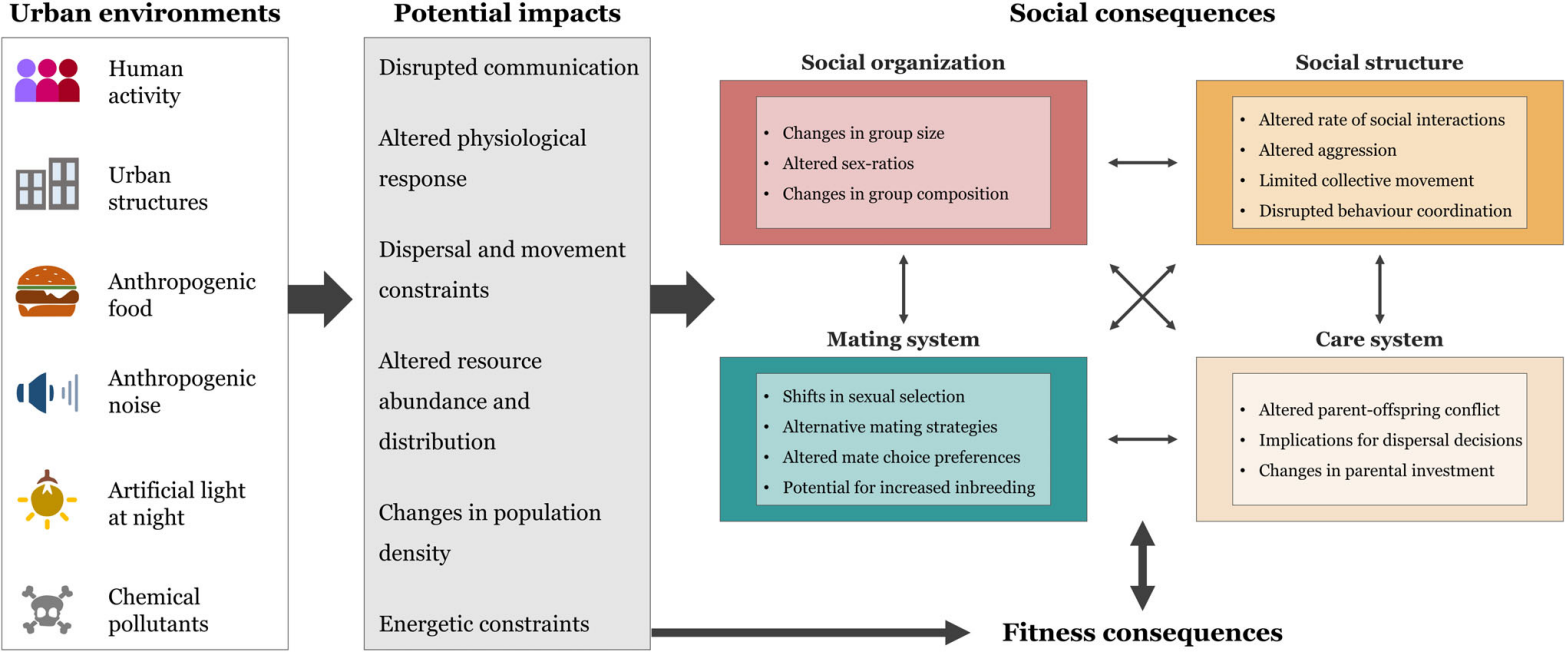
Non‐comprehensive depiction of potential pathways through which urban environments can both directly and indirectly incur social and fitness consequences. The social consequences are grouped according to the four components of an animal's social system (Kappeler, 2019). The arrows indicate the direction of the impact.

Our review revealed that urbanisation can have a substantial impact on social behaviour (for a summary of outcomes, see Table 1) by directly and indirectly imposing constraints on social systems and potentially shifting the costs and benefits of social interactions through interacting pathways (Fig. 3), or by altering relationships between species. One of the most widespread drivers of behavioural change in urban environments is alterations to an animal's sensory environment (Dominoni *et al*., 2020). Exposure to sensory and chemical pollutants can disrupt effective communication, and it is apparent that urbanisation is broadly shifting communication patterns and having cascading effects on other social behaviours with important fitness consequences. However, many questions remain regarding how other environmental conditions in urban habitats are shaping social systems. While harsh environmental conditions have been implicated in the evolution of alloparental care and cooperative breeding (Lukas & Clutton‐Brock, 2017; Martin *et al*., 2020), and sociality has been proposed as a mechanism to buffer against environmental change (Komdeur & Ma, 2021), we expect that the rapid pace of enviornmental change and culmination of multiple urban stressors in cites will pose a unique challenge to the formation and maintance of complex social groups. Instead, urban environments are likely to promote the formation of social aggregations of conspecifics that are loosely structured, and in which group membership is dynamic and not necessarily dependent on relatedness or strong social bonds (Struller *et al*., 2022). Barriers to movement and limited habitat space in urban areas can pose limitations to dispersal and force ‘less social’ or territorial species into higher rates of social interactions (Lacy & Martins, 2003; Tanner & Jackson, 2012), potentially driving the emergence of dominance hierarchies (Fisher *et al*., 2021) or alternative reproductive strategies if territory defence is too costly (Kokko & Rankin, 2006).

Urban environments enact novel selection pressures that can lead to changes in sexually selected traits, and ultimately enact evolutionary change (Yeh, 2004). However, sexual selection may be unlikely to accelerate adaptations to urban environments (Candolin & Heuschele, 2008), and prior reproductive behaviours can become maladaptive in urban environments, leading to evolutionary traps (Candolin, 2019). While some urban exploiters experience enhanced reproductive success in urban environments (Møller, 2010; Weaving *et al*., 2016; Samuelson *et al*., 2018), animals in urban habitats are generally likely to experience fitness costs (Habib *et al*., 2007; Banks *et al*., 2007; Kaiser *et al*., 2011). Species that rely on social bonds to enhance reproductive success (Silk *et al*., 2009; Schülke *et al*., 2010) or synchronise reproductive behaviour (Brandl *et al*., 2021) will be largely affected, while facultatively social species, or species that are able to shift their social behaviour flexibly according to the social landscape (Taborsky & Oliveira, 2012) should be better able to cope with urbanisation and persist in urban habitats.

### (2) Knowledge gaps, biases, and future research directions

**Fig. 4:**
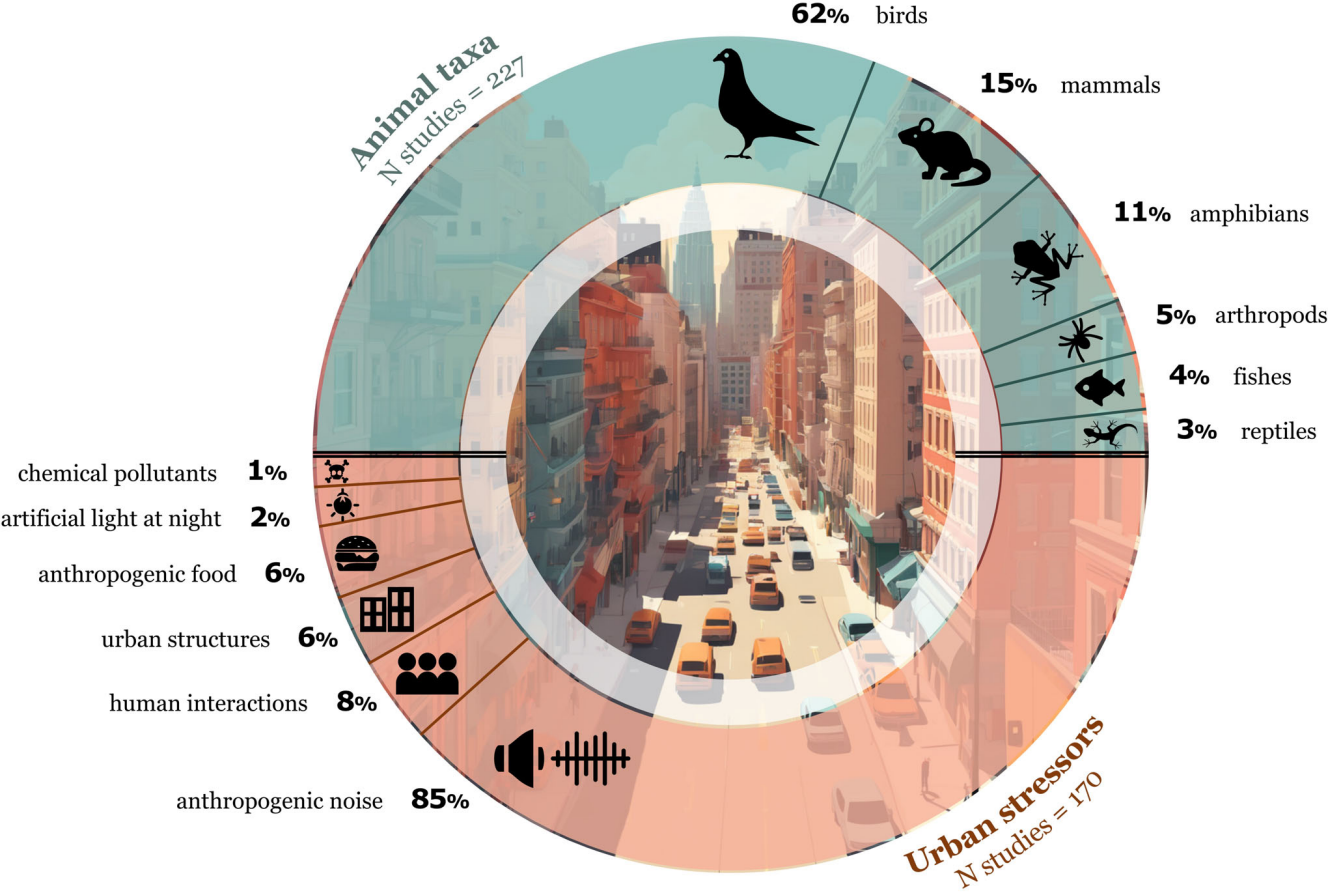
Visual representation of the proportion of studies obtained through our systematic literature search, categorised by taxa (top half, blue) and urban stressor (bottom half, pink). Proportions were rounded to the nearest whole number. Several studies addressed multiple urban stressors (*N* = 13) resulting in a total that exceeds 100% (bottom half).

Animals exhibit obvious variation in social responses to urban conditions, which can be due to species‐specific traits or differences in life history. There is currently a large taxonomic research bias in the overall literature towards endothermic species, particularly birds (Fig. 4), as well as within each urban stressor. For example, studies on anthropogenic noise largely focused on birds (65%, *N* = 94 out of 144 studies), while studies addressing the impact of human activity were primarily performed in social mammals (77%, *N* = 10 out of 13 studies). Birds and mammals can exhibit different behavioural characteristics, dispersal abilities, and energetic demands that influence their response to urbanisation relative to other taxa (e.g. reptiles; French *et al*., 2018), and these biases are limiting our knowledge of how urbanisation is impacting social behaviour. For example, birds commonly exhibit heightened aggression in urban populations, while other species show more varied responses (Table 1), which can be reflective of differences in dispersal abilities mediating the costs of aggressive behaviour and territory defence. While research on model species can provide a useful way to test urban stressors experimentally, research on a broader range of taxa with diverse social systems is necessary to obtain a complete view of how urbanisation is impacting animals and, in turn, ecosystem functioning. For example, eusocial insects exhibit highly structured social organisation and can provide important ecosystem functions (e.g. pollination), yet there is little research on how urbanisation is impacting their social behaviour.

The observed variation in social responses to urbanisation may arise in part from environmental variability within urban habitats. Urban landscapes can be heterogeneous (Cadenasso *et al*., 2007; Grimm *et al*., 2008), and animals living in different geographic urban habitats may be facing different environmental selection pressures and thus exhibit different behavioural responses to urbanisation. For example, the height and density of buildings can differ among cities and parts of the globe, which can differentially impact acoustic communication (Cyr *et al*., 2021), or cities can experience different predation pressures, which can in turn influence grouping behaviour or the conspicuousness of visual displays. Future research should strive to include populations from multiple urban environments or along a gradient of urbanisation to capture this variability. As cities represent a mosaic of different habitats that vary in structural complexity and magnitude of urban stressors, we urge researchers to use metrics to quantify urbanisation (e.g. impermeable surface area, normalised difference vegetation index) and measure ecological parameters (e.g. noise level, light pollution, temperature) instead of classifying habitats on a dichotomy of ‘urban *versus* non‐urban’, which can reveal different significant patterns, and allow researchers to draw parallels more accurately between cities (Szulkin *et al*., 2020; Maraci *et al*., 2022). Along these lines, anthropogenic noise has received considerable research attention and comprised 85% (*N* = 144) of the studies testing specific urban stressors, while studies on human activity, urban structures, artificial light, and chemical pollutants in the context of urbanisation are rare (Fig. 4). While only 13 studies considered more than one urban stressor, animals inhabiting urban areas do not experience stressors in isolation, and it is also important to consider how exposure to multiple urban stressors may have interactive, additive, or antagonistic effects on behaviour [e.g. noise and light pollution (Halfwerk & Jerem, 2021; Cronin *et al*., 2022a; Smit *et al*., 2022), or noise and urban structures (Dowling, Luther & Marra, 2012)]. Experimental approaches that manipulate exposure stressors, or common garden experiments, can provide a useful approach to disentangle the effects of an isolated stressor and how it interacts with added stressors (e.g. McMahon, Rohr & Bernal, 2017).

Social network analysis, a widely used tool to quantify social relationships, can be used to test the impact of urbanisation on social structure directly and allows for comparisons across taxa (Kurvers *et al*., 2014; Krause *et al*., 2015). Studies that investigate how social networks respond to disturbances (e.g. Williams & Lusseau, 2006; Ansmann *et al*., 2012; Lantz & Karubian, 2017) can provide insight into how social structure changes in urban environments, while experimental approaches that add or remove individuals (e.g. Flack *et al*., 2006; Piefke *et al*., 2021), or manipulate environmental conditions (e.g. Firth & Sheldon, 2015; Leu *et al*., 2016; Heinen *et al*., 2022) can allow researchers to identify the drivers shaping social systems in urban habitats. Social network analysis can also be used to quantify how individual attributes, such as social status or personality, influence an individual's social network position (Pike *et al*., 2008; Sih, Hanser & McHugh, 2009), which can influence how an individual experiences urban environments. Furthermore, social network analysis can quantify social behaviour in species that engage in more cryptic social interactions and may revel subtle shifts in social structure in response to urbanisation (e.g. Maune *et al*., 2025).

We need a unified terminology when describing social systems and urbanisation. While mating and parental care behaviours are key components of an animal's social system that can affect other social behaviours, they are rarely discussed in this context, and studies are often missing key words related to social behaviour. Similarly, there is a disconnect in the use of terminology regarding anthropogenic stressors and urbanisation, as evident from our initial literature search. Most of the studies obtained through the backward–forward search were on anthropogenic noise and did not include any terms related to urbanisation in the title, abstract, or key words, despite the fact that some of them were conducted in urban habitats. We suggest that studies addressing any aspect of social behaviour and urbanisation strive to use terms that include ‘social’ and ‘urban’ to make it easier for urban ecologists to identify relevant studies and allow for a greater exchange among disciplines.

Given that many social behaviours are directly linked to reproductive output, it is important to understand the fitness consequences associated with social responses to urbanisation. Identifying whether behavioural responses to urbanisation are adaptive or have associated fitness costs is necessary to form predictions on how species and biotic communities will be impacted as urban areas expand. Translocation and common garden experiments can be used to identify if behavioural changes in urban animals are a result of behavioural plasticity or heritable differences between populations (e.g. Reichard *et al*., 2020). Urbanisation may indeed provide a unique opportunity to test hypotheses regarding social evolution as animal populations are forced to respond and adapt their behaviour to rapidly changing environmental conditions. Furthermore, future research should strive to identify the ecological consequences of behavioural changes to channel resources into ecologically relevant conservation efforts (Wilson *et al*., 2020; Dominoni *et al*., 2020). Relevant topics to explore include the spread of cultural knowledge in urban environments, how urbanisation might increase disease transmission or influence host–parasite relationships by changing the rate and pattern of social interactions, and how urbanisation impacts the social behaviour of highly social species, such as eusocial insects.

From the papers retrieved in our literature search, 92% (*N* = 208) reported a significant impact of urbanisation on some aspect of social behaviour, exemplifying the impact urbanisation can have on animal social systems and interspecific interactions. Nevertheless, this high percentage could also be due to a publication bias towards significant results, and thus, we urge researchers to publish non‐significant results to allow for a more complete understanding of social responses to urbanisation. Given the rate at which urbanisation is increasing (United Nations, Department of Economic and Social Affairs, Population Division, 2019), a proactive approach is needed, and researchers should develop a predictive framework that considers a species' life history, ecology, and individual‐ and population‐level variation in social behaviour. Drawing parallels from studies investigating environmental change or disturbances more broadly (e.g. climate change, wildfires, drought, hunting) can inform *a priori* predictions for how social systems will change in response to urbanisation (Fisher *et al*., 2021; Blumstein *et al*., 2023).

## VII. CONCLUSIONS

(1) Our systematic literature review found that urbanisation is markedly influencing social systems and interspecific interactions (92% of studies found a significant impact), with likely important implications for fitness and survival. We highlight general trends and predictions, as well as gaps and biases in the current scientific literature.
(2) Animals exhibit diverse social responses to urban conditions that can be influenced by a species' life history and specific traits. While this obvious variation makes it difficult to draw overarching conclusions, identifying how specific traits and the evolutionary environment in which they evolved are influencing social responses to urban conditions will provide important insights that can be used to develop predictive frameworks.
(3) Current research is largely biased towards birds, limiting our knowledge of how species with different dispersal abilities, energetic requirements, or social structures are being impacted by urbanisation. For example, birds have a high dispersal ability and commonly exhibit heightened territorial behaviour in urban habitats. Conversely, species that are unable to disperse outside of urban areas may experience higher rates of social encounters and greater territory overlap, which may promote social tolerance and drive the emergence of different social structures, such as dominance hierarchies or social aggregations.
(4) For group‐living species, the culmination of multiple urban stressors in cities will likely destabilise complex social groups by imposing barriers to collective movement and increasing within‐group conflict. Social groups within cities may instead shift to resemble social aggregations where group membership is more dynamic. Facultatively social species, or species with greater social competence, should be better able to cope with urbanisation.
(5) Multiple aspects of social behaviour are directly linked to reproductive output, and different selection pressures in urban habitats may lead to the emergence of alternative mating strategies or change the strength and direction of sexual selection. Ultimately, many species will face fitness consequences, which can in turn further impact social systems.
(6) Future research should take into consideration interactions between multiple stressors, the fitness consequences associated with changes in social interactions, and the addition of other useful approaches, such as social network analysis or common garden experiments. Experimental approaches elucidating the mechanisms driving behavioural changes in urban environments can be used to inform ecologically relevant conservation efforts.
(7) Our review is widely relevant as human‐dominated landscapes are rapidly expanding, and it is becoming increasingly rare that animals will not be exposed to the consequences of human activity. We are enthusiastic that this is a growing field that can be used to guide urban planning and may provide a unique opportunity to further our understanding of social evolution, as animals respond to rapidly changing environmental conditions.

## Data Availability

Data sharing not applicable to this article as no datasets were generated or analysed during the current study.
